# Regio- and enantioselective microbial hydroxylation and evaluation of cytotoxic activity of β-cyclocitral-derived halolactones

**DOI:** 10.1371/journal.pone.0183429

**Published:** 2017-08-24

**Authors:** Marcelina Mazur, Witold Gładkowski, Višnja Gaurina Srček, Kristina Radošević, Gabriela Maciejewska, Czesław Wawrzeńczyk

**Affiliations:** 1 Department of Chemistry, Wrocław University of Environmental and Life Sciences, Wrocław, Poland; 2 Faculty of Food Technology and Biotechnology, University of Zagreb, Zagreb, Croatia; 3 Central Laboratory of the Instrumental Analysis, Wrocław University of Technology, Wrocław, Poland; MJP Rohilkhand University, INDIA

## Abstract

Three β-cyclocitral-derived halolactones, which exhibit antifeedant activity towards storage product pests, were subjected to microbial transformation processes. Among the thirty tested strains of filamentous fungi and yeast, the most effective biocatalysts were *Absidia cylindrospora* AM336, *Mortierella isabellina* AM212 and *Mortierella vinaceae* AM149. As a result of regio- and enantioselective hydroxylation four new oxygenated derivatives were obtained. Regardless of the biocatalyst applied, the δ-iodo- and δ-bromo-γ-lactones were hydroxylated in an inactivated position C-5 of cyclohexane ring. The analogous transformation of chlorolactone was observed in *Mortierella isabellina* AM212 culture but in the case of two other biocatalysts the hydroxy group was introduced at C-3 position. All obtained hydroxylactones were enantiomerically pure (ee = 100%) or enriched (ee = 50%). The highest enantioselectivity of hydroxylation was observed for *M*. *isabellina* AM212. The cytotoxic activity of halolactones was also examined by WST-1 assay wherein tested compounds did not exhibit significant effect on the viability of tumor HeLa and normal CHO-K1 cells.

## Introduction

The hydroxylation of “inactivated” position in substrate structure leading to enantiomerically pure or enriched products arouse great interest but it is difficult to carry out by traditional chemical synthesis. One of the most efficient methods of incorporation the hydroxy group into organic molecule is the application of microorganisms [1,2,3]. It is also worth to notice that processes catalyzed by the microorganisms have less harmful impact on natural environment than methods of organic synthesis [4]. The biotransformations catalyzed by whole cells are convenient and useful biotechnological tools, especially taking into consideration that majority of hydroxylation processes are mediated by membrane enzymes hence they cannot be easily purified. Different microorganisms can be applied to the enantioselective hydroxylation processes [5, 6]. In our present work we tested thirty strains of filamentous fungi and yeast from seventeen different genus. Three strains of filamentous fungi from *Absidia* and *Mortierella* genus were found to be efficient catalysts. The different *Mortierella* species are investigated as a potent producer of arachidonic acid but only few examples of their use for biohydroxylation are described in literature [7–9]. More examples of hydroxylation can be found for *Absidia* genus [10–12]. Different *Absidia* strains are able to transform biologically active compounds [13,14], including those with steroid skeleton [15,16] or lactone ring [17–21].

In this work we present the hydroxylation of β-cyclocitral-derived halolactones by three fungal strains (*Absidia cylindrospora* AM336, *Mortierella isabellina* AM212 and *Mortierella vinaceae* AM149). Parent compounds exhibit antifeedant activity [22] hence it is important to establish the possible metabolic pathway of those compounds in natural environment. Biotransformations of halolactones involving the hydroxylation reaction which entails the increase of their polarity is essential during their further biodegradation. Additionally the previous reports revealed the anticancer potential of some δ-halo-γ-lactones [23, 24] which prompted us to check also the cytotoxic activity of halolactones derived from β-cyclocitral.

## Material and methods

### Analysis

The progress of transformations as well as the purity of isolated products were checked by Thin Layer Chromatography (TLC, silicagel couted aluminium plates, DC-Alufolien Kieselgel 60 F_254_, Merck) and gas chromatography (GC). GC analysis was performed on a Agilent Technologies 6890N instrument using Agilent DB-17 capillary column ((50%-phenyl)-methylpolysiloxane 30 m × 0.25 mm × 0.25 μm) and the hydrogen as the carrier gas. The temperature programme was as follows: injector 250°C, detector (FID) 300°C, column temperature: 80°C (1min), 80–200°C (rate 20°C min^−1^), 200–300°C (rate 30°C min^−1^), 300°C (2 min). The enantiomeric excesses of biotransformation products were calculated on the basis of GC analysis using CP Chirasil-Dex CB column (25 m x 0.25 mm x 0.25 μm) at the following conditions: injector 200°C, detector (FID) 250°C, column temperature: 75°C (hold 1 min), 75–190°C (rate 0.42°C min^-1^), 190°C (hold 1 min), 190–200 (rate 0.5°C min^-1^), 200°C (hold 10 min).

The biotransformation products were purified by column chromatography on silica gel (Kieselgel 60, 230–400 mesh, Merck).

The NMR spectra (^1^H, ^13^C NMR and correlation spectra: ^1^H-^1^H COSY, ^1^H-^13^C HMQC, ^1^H-^13^C HMBC) were recorded in a CDCl_3_ solution on a Brüker Avance II 600 MHz spectrometer. Residual solvent signals (δ_H_ = 7.26, δ_C_ = 77.0) were used as references for chemical shifts.

IR spectra were determined using Mattson IR 300 Thermo Nicolet spectrophotometer. The melting points (uncorrected) were determined on a Boetius apparatus. Optical rotations were measured on Jasco P-2000 digital polarimetr (version with iRM controller).

High resolution mass spectra (HRMS) were recorded using electron spray ionization (ESI) technique on spectrometer Waters ESI-QTOF Premier XE.

### Substrates for biotransformation

Three racemic lactones containing different halogen atoms: 1-iodomethyl-2,2,6-trimethyl-9-oxabicyclo [4.3.0]nonan-8-one (**1**), 1-bromomethyl-2,2,6-trimethyl-9-oxabicyclo[4.3.0]nonan-8-one (**2**) and 1-chloromethyl-2,2,6-trimethyl-9-oxabicyclo[4.3.0]nonan-8-one (**3**) were synthesized from β-cyclocitral in four steps chemical synthesis, according to the procedure described earlier [22].

Here we present the spectral data of those compounds in order to compare them with the spectra of corresponding products of microbial transformations.

1-Iodomethyl-2,2,6-trimethyl-9-oxabicyclo [4.3.0]nonan-8-one (**1**)

mp = 104–107°C; ^**1**^**H NMR** (600 MHz, CDCl_3_)δ: 1.10 and 1.17 (two s, 6H, (CH_3_)_2_C<), 1.34 (s, 3H, CH_3_-6), 1.38–1.75 (m, 6H, CH_2_-4, CH_2_-5, CH_2_-6), 2.21 and 3.26 (two d, *J* = 17.5 Hz, 2H, CH_2_-7), 3.52 and 3.84 (two d, *J* = 11.8 Hz, 2H, CH_2_-10); ^**13**^**C NMR** (151 MHz, CDCl_3_) δ: 6.09 (C-10), 17.26 (C-4), 21.74 (CH_3_-6), 25.53 and 27.63 ((*C*H_3_)_2_C<), 38.18 (C-2), 38.31 (C-3), 39.86 (C-5), 42.39 (C-6), 47.84 (C-7), 88.02 (C-1), 175.62 (C-8); **IR** (KBr, cm^-1^): 2928 (s), 1767 (s), 1245 (m), 633 (w);

1-Bromomethyl-2,2,6-trimethyl-9-oxabicyclo [4.3.0]nonan-8-one (**2**)

mp = 156–161°C; ^**1**^**H NMR** (600 MHz, CDCl_3_) δ: 1.07, 1.15 (two s, 6H, (CH_3_)_2_C<), 1.37 (s, 3H, CH_3_-6), 1.38 (m, 1H, one of CH_2_-3), 1.42–1.48 (m, 2H, one of CH_2_-4 and one of CH_2_-5), 1.53 (m, 1H, one of CH_2_-4), 1.62–1.71 (m, 2H, one of CH_2_-3 and one of CH_2_-5), 2.17 and 3.25 (two d, *J* = 17.5 Hz, 2H, CH_2_-7), 3.73 and 3.97 (two d, *J* = 11.9 Hz, 2H, CH_2_-10); ^**13**^**C NMR** (151 MHz, CDCl_3_) δ: 17.42 (C-4), 21.90 (CH_3_-6), 25.34 and 27.43 ((*C*H_3_)_2_C<), 35.15 (C-10), 38.07 (C-2), 38.57 (C-3), 40.08 (C-5), 42.07 (C-6), 47.85 (C-7), 88.67 (C-1), 175.93 (C-8); **IR** (KBr, cm^-1^): 2976 (s), 1767 (s), 1243 (s), 656 (m);

1-Chloromethyl-2,2,6-trimethyl-9-oxabicyclo [4.3.0]nonan-8-one (**3**)

mp = 97–101°C; ^**1**^**H NMR** (600 MHz, CDCl_3_) δ: 1.05, 1.14 (two s, 6H, (CH_3_)_2_C<), 1.34 (m, 1H, one of CH_2_-3), 1.36 (s, 3H, CH_3_-6), 1.42–1.48 (m, 2H, one of CH_2_-4 and one of CH_2_-5), 1.53 (m, 1H, one of CH_2_-4), 1.61–1.70 (m, 2H, one of CH_2_-3 and one of CH_2_-5), 2.16 and 3.14 (two d, *J* = 17.4 Hz, 2H, CH_2_-7), 3.88 and 4.05 (two d, *J* = 12.6 Hz, 2H, CH_2_-10); ^**13**^**C NMR** (151 MHz, CDCl_3_) δ: 17.50 (C-4), 21.93 (CH_3_-6), 25.08 and 27.38 (*C*H_3_)_2_C<), 37.55 (C-2), 38.61 (C-3), 40.10 (C-5), 41.74 (C-6), 46.93 (C-10), 47.82 (C-7), 89.34 (C-1), 176.08 (C-8); **IR** (KBr, cm^-1^): 2934 (s), 1773 (s), 1254 (s), 731 (m);

### Microbial transformations

Thirty strains of filamentous fungi and yeasts used in this work came from the collection of the Institute of Biology and Botany, Wrocław Medical University (*Absidia coerulea* AM93, *Absidia glauca* AM254, *Absidia cylindrospora* AM336, *Armillaria mellea* AM296, *Beauveria bassiana* AM278, *Chaetomium* sp. AM432, *Fusarium avenaceum* AM12, *Fusarium culmorum* AM10, *Fusarium culmorum* AM196, *Fusarium scirpi* AM199A, *Fusarium solani* AM203, *Fusarium tricinctum* AM16, *Laetiporus sulphurens* AM524, *Mortierella isabellina* AM212, *Mortierella vinaceae* AM149, *Mucor circinelloides* AM385, *Nigrospora oryzae* AM8, *Penicillium camembertii* AM83, *Penicillium chermesinum* AM113, *Penicillium frequentans* AM351, *Penicillium lilacinum* AM111, *Penicillium vermiculatum* AM81, *Pholiota aurivella* AM522, *Rhodotorula marina* AM77, *Rhodotorula rubra* AM82, *Saccharomyces cerevisiae* AM464, *Spicaria fusispora* AM136, *Syncephalastrum racemosum* AM105, *Trametes versicolor* AM536) and from the collection of the Department of Phytopatology, Kraków Agricultural University (*Cenangium ferruginosum* AR56). Cultivation of microorganisms was carried out on Sabouraud agar slants of following composition: aminobac (5.0 g), peptone K (5.0 g), glucose (40.0 g) in distilled water (1 L). The microorganisms were cultivated at 28°C and stored in refrigerator at 4°C.

### Screening procedure

The strains were cultivated at 25°C in 300 mL Erlenmeyer flasks containing 50 mL of medium (30.0 g glucose, 5.0 g peptone K, 5.0 g aminobac in 1 L of distilled water). After 4 days of growth 10 mg of iodolactone (**1**) dissolved in 1 mL of acetone were added to the shaken cultures (150 rpm). The incubation was continued for 15 days. After 1, 2, 3, 6, 9, 12 and 15 days of incubation, the products were extracted with methylene chloride and analyzed by TLC and GC. Three strains of fungi *(A*. *cylindrospora* AM336, *M*. *isabellina* AM212 and *M*. *vinaceae* AM149) were able to transform the iodolactone (**1**). For selected strains the screening experiments with bromo- (**2**) and chlorolactone (**3**) as substrates were carried out as described above.

### Biotransformation

The screening experiments allowed as to choose three strains of fungi capable to transform iodo-, bromo- and chlorolactone (**1**–**3**) (*A*. *cylindrospora* AM336, *M*. *isabellina* AM212 and *M*. *vinaceae* AM149). For isolation and identification of products, the biotransformation were performed in multiplied scale. Selected strains were cultivated in 10 Erlenmeyer flasks with 50 mL of medium to which 100 mg of substrate dissolved in acetone was totally added (reaction conditions the same as described in screening procedure). After the optimum time for each biotransformation process the products were extracted two times with methylene chloride (50 mL for each flask). The organic layers were pooled, dried over anhydrous MgSO_4_ and the solvent was evaporated *in vacuo*. The transformation products were separated and purified by column chromatography (hexane:acetone from 7:1 to 4:1). As a result of biotransformation of (**1**) in *A*. *cylindrospora* AM336 culture, 27 mg (26% yield) of hydroxyiodolactone (**4**) was obtained, for *M*. *isabellina* AM212 and *M*. *vinaceae* AM149 21 mg (20% yield) and 19 mg (18% yield), were isolated respectively. Products of bromolactone (**2**) transformations were isolated in 14% yields (15 mg) using *A*. *cylindrospora* AM336, in 24% yield (20 mg) using *M*. *isabellina* AM212 and in 16% yield (17 mg) using *M*. *vinaceae* AM149. Hydroxylated metabolites of chlorolactone (**3**) were obtained using *A*. *cylindrospora* AM336 culture in 30% yield (32 mg), using *M*. *vinaceae* AM149 in 31% yield (33 mg) and using *M*. *isabellina* AM212 in 49% yield (52 mg). The physical and spectral data of biotransformation products are given below.

(+)-5-Hydroxy-1-iodomethyl-2,2,6-trimethyl-9-oxabicyclo [4.3.0]nonan-8-one (**4**)

White crystals, mp = 168–172°C; $\alpha_{D}^{25}$ = +30.13 (c = 0.45, CH_2_Cl_2_, ee = 100%); ^**1**^**H NMR** (600 MHz, CDCl_3_) δ: 1.09 and 1.17 (two s, 6H, (CH_3_)_2_C<), 1.26 (s, 3H, CH_3_-6), 1.36 (ddd, *J* = 13.9, 7.4 and 4.3 Hz, 1H, one of CH_2_-3), 1.61–1.65 (m, 2H, CH_2_-4), 1.71 (m, 1H, one of CH_2_-3), 2.72 and 3.16 (two d, *J* = 17.8 Hz, 2H, CH_2_-7), 3.39 (dd, *J* = 9.4, 6.6 Hz, 1H, H-5), 3.55 and 3.80 (two d, *J* = 12.0 Hz, 2H, CH_2_I); ^**13**^**C NMR** (151 MHz, CDCl_3_) δ: 5.87 (C-10), 13.47 (CH_3_-6), 25.28 and 27.52 ((*C*H_3_)_2_C<), 26.44 (C-4), 36.72 (C-3), 38.15 (C-2), 44.64 (C-7), 48.27 (C-6), 75.11 (C-5), 88.91 (C-1), 175.54 (C-8); **IR** (KBr, cm^-1^): 3549 (s), 3479 (s), 2981 (s), 1758 (s), 1259 (s), 622 (m). **HRMS** (ESI-TOF) m/z [M+H]^+^ calcd for C_12_H_19_IO_3_ 339.0457; found 339.0449.

(+)-5-Hydroxy-1-bromomethyl-2,2,6-trimethyl-9-oxabicyclo [4.3.0]nonan-8-one (**5**)

White crystals, mp = 165–169°C; $\alpha_{D}^{25}$ = +30.88 (c = 0.5, CH_2_Cl_2_, ee = 95%); ^**1**^**H NMR** (600 MHz, CDCl_3_) δ: 1.07 and 1.14 (two s, 6H, (CH_3_)_2_C<), 1.29 (s, 3H, CH_3_-6), 1.39 (dt, *J* = 13.4 and 3.1 Hz, 1H, one of CH_2_-3), 1.61–1.66 (m, 2H, CH_2_-4), 1.71 (m, 1H, one of CH_2_-3), 2.67 and 3.18 (two d, *J* = 17.9 Hz, 2H, CH_2_-7), 3.41 (dd, *J* = 9.1 and 6.9 Hz, 1H, H-5), 3.76 and 3.94 (two d, *J* = 12.1 Hz, 2H, CH_2_Br); ^**13**^**C NMR** (151 MHz, CDCl_3_) δ: 13.54 (CH_3_-6), 25.10 and 27.20 ((*C*H_3_)_2_C<), 26.54 (C-4), 35.26 (C-10), 36.94 (C-3), 38.13 (C-2), 44.77 (C-7), 47.88 (C-6), 75.23 (C-5), 89.67 (C-1), 175.88 (C-8); **IR** (KBr, cm^-1^): 3485 (s), 2988 (s), 1762 (s), 1259 (s), 661 (m). **HRMS** (ESI-TOF) m/z [M+H]^+^ calcd for C_12_H_19_BrO_3_ 291.0596; found 291.0599.

(+)-3-Hydroxy-1-chloromethyl-2,2,6-trimethyl-9-oxabicyclo [4.3.0]nonan-8-one (**6**)

mp = 152–157°C, $\alpha_{D}^{25}$ = +1.93 (c = 0.73, CH_2_Cl_2_, ee = 67%); ^**1**^**H NMR** (600 MHz, CDCl_3_) δ: 0.99 and 1.23 (two s, 6H, (CH_3_)_2_C<), 1.37 (s, 3H, CH_3_-6), 1.54–1.65 (m, 3H, CH_2_-4 and one of CH_2_-5), 1.72 (m, 1H, one of CH_2_-5), 2.13 and 3.18 (two d, *J* = 17.4 Hz, 2H, CH_2_-7), 3.63 (dd, *J* = 12.0 and 6.0 Hz, 1H, H-3), 3.93 and 4.08 (two d, *J* = 12.7 Hz, 2H, CH_2_Cl); ^**13**^**C NMR** (151 MHz, CDCl_3_)δ; 16.34 and 22.40 ((*C*H_3_)_2_C<), 21.82 (CH_3_-6), 26.22 (C-4), 37.86 (C-5), 41.84 (C-6), 42.93 (C-2), 46.78 (C-10), 47.92 (C-7), 73.48 (C-3), 90.70 (C-1), 175.70 (C-8);); **IR**(KBr, cm^-1^): 3508 (s), 2978 (s), 1752 (s), 1269 (s) 731 (m). **HRMS** (ESI-TOF) m/z [M+H]^+^ calcd for C_12_H_19_ClO_3_ 247.1101; found 247.1114.

(+)-5-Hydroxy-1-chloromethyl-2,2,6-trimethyl-9-oxabicyclo [4.3.0]nonan-8-one (**7**)

White crystals, mp = 165–170°C, $\alpha_{D}^{25}$ = +4.33 (c = 1.3, CH_2_Cl_2_, ee = 66%); ^**1**^**H NMR** (600 MHz, CDCl_3_) δ: 1.05 and 1.12 (two s, 6H, (CH_3_)_2_C<), 1.29 (s, 3H, CH_3_-6), 1.37 (m, 1H, one of CH_2_-3); 1.59–1.77 (m, 3H, one of CH_2_-3 and CH_2_-4), 2.66 and 3.06 (two d, *J* = 17.9 Hz, 2H, CH_2_-7), 3.41 (dd, *J* = 8.7, 7.5 Hz, 1H, H-5), 3.91 and 4.01 (two d, *J* = 12.7 Hz, 2H, CH_2_Cl); ^**13**^**C NMR** (151 MHz, CDCl_3_) δ: 13.64 (CH_3_-6), 24.87 and 27.15 ((*C*H_3_)_2_C<), 26.54 (C-4), 36.97 (C-3), 37.58 (C-2), 44.68 (C-7), 47.22 (C-10), 47.51 (C-6), 75.17 (C-5), 90.41 (C-1), 176.10 (C-8); **IR** (KBr, cm^-1^): 3491 (s), 2981 (s), 1756 (s), 1261 (s), 733 (m). **HRMS** (ESI-TOF) m/z [M+H]^+^ calcd for C_12_H_19_ClO_3_ 247.1101; found 247.1113.

### Cytotoxic activity

The effect of three halolactones (**1, 2, 3**) on proliferation of HeLa (provided from Institute Rudjer Boskovic, Zagreb, Croatia) and CHO-K1 (ATCC CCL-61) cells was examined by WST-1 assay. Cells were cultured in DME medium supplemented with 10% (v/v) FBS in the incubator with 5% CO_2_ and humidified atmosphere at 37°C. HeLa and CHO-K1 cells from exponential growth phase were seeded in 96-well plates at initial concentration of 5 × 10^−4^ cells per well in 100 μL of media. After 24 h of inoculation, culture media was replaced by fresh one containing 100 and 200 μM of tested lactones. Stock solutions of lactones (**1, 2, 3**) were prepared in DMSO, and filtered through 0.22 filters. After 72 h of treatment, 10 μL of WST-1 reagent was added to each well and cells were incubated for further 4 h. Absorbance was measured at 450 nm on the microplate reader (Tecan, Switzerland). The experiments were performed in triplicate and data were expressed as means ± S.D. of percentages of treated cells versus control (untreated cells).

## Results and discussion

**Fig 1:**
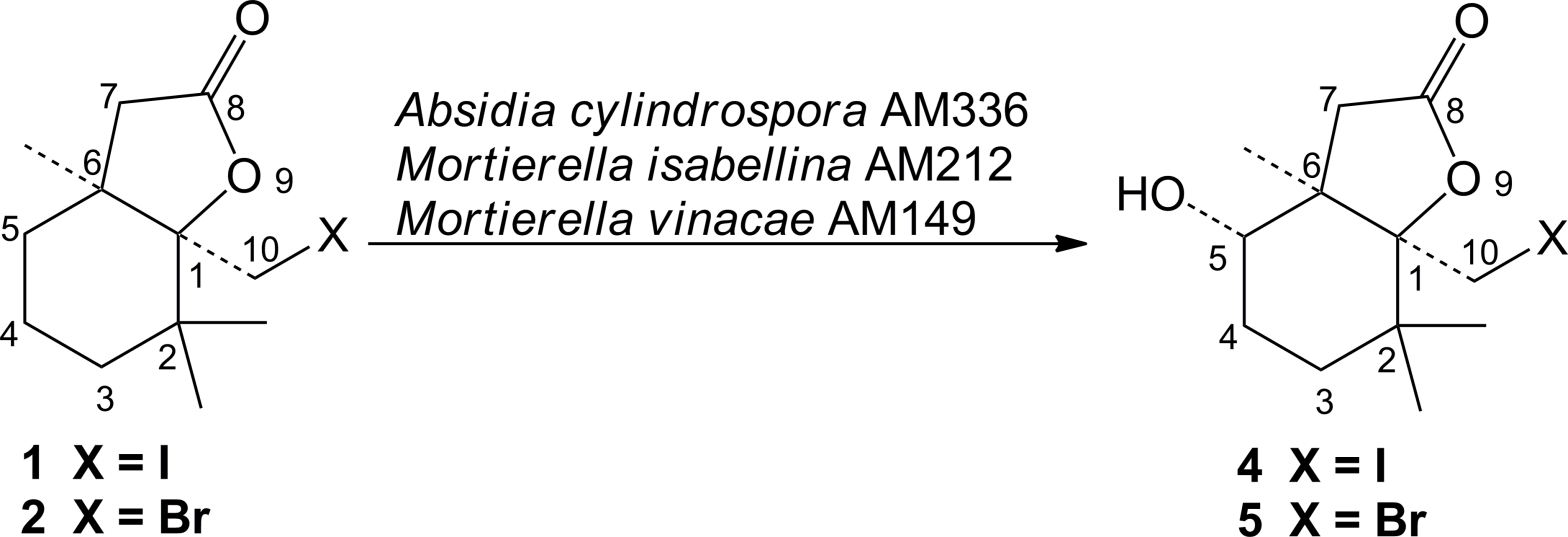
Biohydroxylation of iodo- and bromolactones (1 and 2).

The metabolism of three β-cyclocitral-derived halolactones which exhibit antifeedant activity towards storage product pests [22] was investigated in fungal strains cultures. Preliminary screening procedure was carried out for iodolactone (**1**). Thirty strains of filamentous fungi and yeast, listed in the experimental part, were checked to select efficient biocatalyst. Among them, three strains: *Absidia cylindrospora* AM336, *Mortierella isabellina* AM212 and *Mortierella vinaceae* AM149 were able to modify the iodolactone structure (**Fig 1**). In the next step these selected microorganisms were tested for their ability are to transform also the bromo- and chlorolactone (**2** and **3**). The results of screening experiments as the composition of product mixtures during the biotransformation processes are presented in **Table 1**. The *Mortierella vinaceae* AM149 was found to transform all substrates at the highest rate. On the contrary, the longest time of transformation was observed in the case of found for processes catalyzed by *Absidia cylindrospora* AM336 and even after 15 days substrates were not consumed completely.

**Table 1:** The composition (in % according to GC) of the products mixtures in the screening biotransformations of halolactones 1, 2 and 3.

Time of incubation (days)	*A*. *cylindrospora*AM336		*M*. *isabellina* AM212		*M*. *vinaceae* AM149	
	**Substrate 1**	**Product 4**	**Substrate 1**	**Product 4**	**Substrate 1**	**Product 4**
1	100	0	57	43	49	51
2	100	0	44	56	32	68
3	74	26	36	64	18	82
6	59	41	21	79	0	100
9	46	54	13	87	0	100
12	23	77	4	96	0	100
15	18	82	0	100	0	100
	**Substrate 2**	**Product 5**	**Substrate 2**	**Product 5**	**Substrate 2**	**Product 5**
1	100	0	89	11	52	48
2	91	9	63	37	23	77
3	79	21	34	66	17	83
6	62	38	18	82	6	94
9	42	58	11	89	3	97
12	22	78	0	100	0	100
15	14	86	0	100	0	100
	**Substrate 3**	**Product 6**	**Substrate 3**	**Product 7**	**Substrate 3**	**Product 6**
1	98	2	65	35	79	21
2	91	9	54	46	51	49
3	85	15	39	61	25	75
6	69	31	22	78	3	97
9	42	58	6	94	0	100
12	27	73	0	100	0	100
15	13	87	0	100	0	100

Based on the data obtained in the screening experiments, the biotransformation processes in multiplied scales were carried out in order to isolate and determine the structures of obtained products. Because of intensification of metabolites production in the last days of processes, we decided to shorten the transformation time to simplify the isolation procedure. The optimal biotransformation time for *Absidia cylindrospora* AM336 was twelve days, for *Mortierella isabellina* AM212 and *Mortierella vinaceae* AM149 nine and three days, respectively.

Biotransformation of iodolactone (**1**) in the culture of all selected fungal strains resulted in the formation of the same (+)-enantiomer of product, but its enantiomeric excess depended on the biocatalyst (**Table 2**). Pure enantiomer of product **4** was produced by *M*. *isabellina* AM212. The process catalyzed by *A*. *cylindrospora* AM336 was the least enantioselective (ee of product—50%). The isolated yields of the product were rather moderate and did not exceed 26% as obtained in *A*. *cylindrospora* AM336—catalyzed transformation. The structure of product was established on the basis of its spectroscopic data (S1–S3 Figs). IR spectrum of lactone **4** showed the presence of absorption band at 1758 cm^-1^ characteristic for five membered lactone ring and O-H band stretching vibration at 3479 cm^-1^ confirming the introduction of the hydroxy group. The location of this functional group was determined by comparison of the ^1^H NMR spectrum of product **4** and parent structure **1**. On ^1^H NMR spectrum of the hydroxyiodolactone (**4**) characteristic doublets from C*H*_*2*_-10 groups can be observed. The presence of those signals excluded the dehalogenation process. Incorporation of hydroxy group into the molecule was additionally confirmed by the deshielding effect of oxygen from OH function which resulted in the shift of characteristic doublet of doublets from *H*-5 proton to 3.39 ppm. Coupling constant value between one of the C*H*_2_-4 protons and *H*-5 proton (*J* = 9.4 Hz) suggested the axial orientation of the latter and consequently equatorial location of hydroxy group. The crucial was HMBC correlation, with allowed to confirm the exact location of hydroxy group. The coupling between carbon *C*-7, *C*H_3_-6 and proton *H*-5, undoubtedly confirms the introduction of the OH group at *C*-5.

**Table 2:** The enantiomeric excess (ee %) of obtained biotransformation products.

Strain	Product	Ee	Product	ee	Product	ee
*A*. *cylindrospora*AM336	*(+)*-**4**	50%	*(+)*-**5**	76%	*(+)*-**6**	52%
*M*. *isabellina* AM212	*(+)-***4**	100%	*(+)*-**5**	95%	*(+)*-**7**	66%
*M*. *vinaceae* AM149	*(+)-***4**	90%	*(+)*-**5**	91%	*(+)*-**6**	67%

Strong similarities can be noticed for biotransformation of bromolactone (**2**) (**Fig 1**). In those processes also the enantiomerically enriched (+)-isomer of hydroxybromolactone (**5**) was obtained as the only product. The highest enantiomeric excess (ee = 95%) was determined for the product obtained in *M*. *isabellina* AM212 culture as in the case of transformation of iodolactone (**1**). Analogically, the lowest ee value of hydroxybromolactone (**5**) (ee = 76%) was observed when *Absidia cylindrospora* AM336 was used as biocatalyst (**Table 2**).

The spectroscopic data of the substrate (**2**) and product of biotransformation (**5**) (S4–S6 Figs) exhibited differences analogical to those described above for hydroxyiodolactone (**4**). The presence of absorption bands at 1762 cm^-1^ and 3549 cm^-1^ on IR spectrum proved the retention of the γ-lactone moiety and incorporation of hydroxy function. On ^1^H NMR spectrum at 3.41 ppm the characteristic doublet of doublets from proton *H*-5 is present. The coupling constant *J* = 9.1 Hz found in this signal confirmed axial orientation of proton *H*-5 and as a consequence the equatorial position of hydroxy group. Further, the coupling between the proton *H*-5 and carbon atoms *C*-7 and *C*H_3_-6 on HMBC spectrum, confirmed the incorporation of OH group into *C*-5 position.

**Fig 2:**
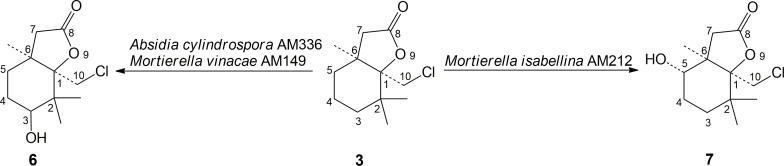
Biohydroxylation of chlorolactone (3).

The products of biohydroxylations of chlorolactone **3** (**Fig 2**) exhibited lower enantiomeric excess (52–67%) compare to iodo- and bromoderivatives (**Table 2**). In all experiments (+)*-*isomer of product **6** or **7** was the major one. The hydroxychlorolactone **7** obtained in *Mortierella isabellina* AM212 culture was the product of biohydroxylation of substrate at *C*-5. The spectral data (S7–S9 Figs) were comparable to those described above for products **4** and **5**. Evident similarities like absorption band at 1756 cm^-1^ and 3491 cm^-1^ on IR spectrum and correlation between proton *H*-5 and carbon atoms *C*H_3_-6 and *C*-7 on HMBC spectrum were found. The equatorial orientation of OH group was confirmed on the basis of the signal shape and constant coupling values (*J* = 8.7 and 7.5 Hz) of proton *H*-5. Taking into consideration numerous similarities in the biotransformation of δ-iodo-γ-lactone **1** and δ-bromo-γ-lactone **2**, the transformation of δ-chloro-γ-lactone **3** in *Absidia cylindrospora* AM336 and *Mortierella vinaceae* AM149 cultures resulted in the formation of unexpected product **6** (**Fig 2**). The analysis of its spectral data revealed that this time the substrate **3** was hydroxylated at *C*-3 position of cyclohexane ring (S10–S12 Figs). On HMBC spectrum the crucial confirmation was coupling between *C*-3 shifted to δ = 73.48 ppm and protons of two geminal methyl groups. Significant difference in chemical shifts can be noticed between the ^1^H NMR spectra of products **6** and **7**. As a result of deshielding effect of oxygen from OH group the signal from the protons of one of methyl group in *gem*-dimethyl substituent was shifted to the lower field (1.23 ppm) for product of *C*-3 hydroxylation comparing with 1.12 ppm of hydroxylactone **7**. The axial orientation of proton *H*-3 and consequently equatorial position of hydroxy group is confirmed by constant coupling values (*J* = 12.0 Hz) found for *H*-3 signal. The product of biotransformation of chlorolactone **3** were obtained in high isolated yields within the range from 30% in the case of *Absidia cylindrospora* AM336 to even almost 50% in the case of *Mortierella isabellina* AM212 culture.

The most popular biocatalytic process used to obtain the optically active alcohols is asymmetric reduction of prochiral ketones [25–27] and lipase-catalyzed kinetic resolution of racemic alcohols [28, 29]. In both cases the basic limitation is the presence of carbonyl or hydroxy function already in the defined place in a substrate molecule. In literature we can find numerous examples of enantioselective hydroxylation of inactivated position in steroid skeleton [6, 14, 30, 31] and terpenes [13, 32]. Nevertheless, the regio- and stereoselective incorporation of hydroxy function into smaller non-functionalized molecules is still a challenge [33]. As reported in previous studies, the fungal metabolism of bicyclic lactones with cyclohexane system can be complex and often engages the oxidation reactions. Genus *Absidia* is one of the most potent biological systems involving different metabolic pathways in transformations processes. The ability of *Absidia cylindrospora* for catalyzing hydroxylation reactions, various dehalogenation reactions or epoxydation of double bond is well documented. Regardless of the biocatalyst the hydroxy group is commonly incorporated at a tertiary carbon atom [20, 21, 34] or into alkyl substituents [7, 35]. For unsaturated lactones the formation of epoxy products can be observed [20]. Alternatively, halolactones can be subjected to different dehalogenation reactions. These processes lead mainly to the products of hydrolytic dehalogenation in which halogen atom is replaced by hydroxy group [36] or unsaturated lactones as the dehydrohalogenation products [8, 37]. In our work we obtained products of enantioselective hydroxylation of secondary carbon atoms in cyclohexane moiety. All obtained products are not reported previously and what is also worth to notice, unlike C-2, C-3 and C-4 hydroxylations the C-5 biohydroxylation was not described in literature for this group of compounds. Additionally, retention of halogen atom as additional reactive center can be important in context of their possible use as multifunctional chiral building blocks.

To confirm possible antiproliferative effect of halolactones (**1, 2, 3**), we evaluated their *in vitro* cytotoxicity towards HeLa and CHO-K1 cell lines by WST-1 assay. Obtained results expressed as a percentage of cell viability are presented in **Table 3**. Tested compounds did not show the significant decrease of cell viability in both cell lines even at 200 μM concentration. Nevertheless, the obtained cytotoxicity results are valuable as a guide how to direct the synthesis of similar analogues with improved biological properties.

**Table 3:** Effect of halolactones (1, 2, 3) on HeLa and CHO cell viability, determined by the WST-I assay. The viability (%) was expressed as percentage of treated cells versus control cells.

Compound	Concentration			
	HeLa		CHO-K1	
	100μM	200μM	100μM	200μM
**1**	98.89±5.58	87.50±3.54	97.08±10.61	100.85±1.92
**2**	97.69±1.78	90.13±4.00	100.08±6.82	100.85±7.29
**3**	97.05±2.65	92.66±4.96	97.19±3.48	102.77±3.80

## Conclusion

Among thirty strains of filamentous fungi and yeast three of them: *Absidia cylindrospora* AM336, *Mortierella isabellina* AM212 and *Mortierella vinaceae* AM149 were able to incorporate the hydroxy function into substrate structure. All biotransformation processes were regio- and enantioselective and new hydroxyhalolactones were isolated and fully characterized by spectroscopic data. In all four products the hydroxy group was incorporated in inactivated methylene carbon atom of cyclohexane moiety. The most significant differences between the biocatalysts can be observed during the transformation of chlorolactone (**3**). *Absidia cylindrospora* AM336 and *Mortierella vinaceae* AM149 exhibited the tendency of *C*-3 hydroxylation and *Mortierella isabellina* AM212 strain hydroxylated *C*-5 carbon atom regardless of the halogen type in substrate structure. Presented biohydroxylations is efficient method to obtained enantiomerically pure or enrich hydroxyhalolactones—the multifunctional compounds that potentially can be used as the chiral building blocks.

## Supporting information

S1 Fig ^1^H NMR and ^13^C NMR spectra of hydroxyiodolactone 4. (PDF)

S2 Fig IR spectrum of hydroxyiodolactone 4. (PDF)

S3 Fig HRMS spectrum of hydroxyiodolactone 4. (PDF)

S4 Fig ^1^H NMR and ^13^C NMR spectra of hydroxybromolactone 5. (PDF)

S5 Fig IR spectrum of hydroxybromolactone 5. (PDF)

S6 Fig HRMS spectrum of hydroxybromolactone 5. (PDF)

S7 Fig ^1^H NMR and ^13^C NMR spectra of hydroxychlorolactone 7. (PDF)

S8 Fig IR spectrum of hydroxychlorolactone 7. (PDF)

S9 Fig HRMS spectrum of hydroxychlorolactone 7. (PDF)

S10 Fig ^1^H NMR and ^13^C NMR spectra of hydroxychlorolactone 6. (PDF)

S11 Fig IR spectrum of hydroxychlorolactone 6. (PDF)

S12 Fig HRMS spectrum of hydroxychlorolactone 6. (PDF)
