# Supplementary figures and images for: Regio- and enantioselective microbial hydroxylation and evaluation of cytotoxic activity of β-cyclocitral-derived halolactones
Source: PLoS One. 2017 Aug 24;12(8):e0183429. doi: 10.1371/journal.pone.0183429 (PMC5570294; doi:10.1371/journal.pone.0183429)

# Product 4

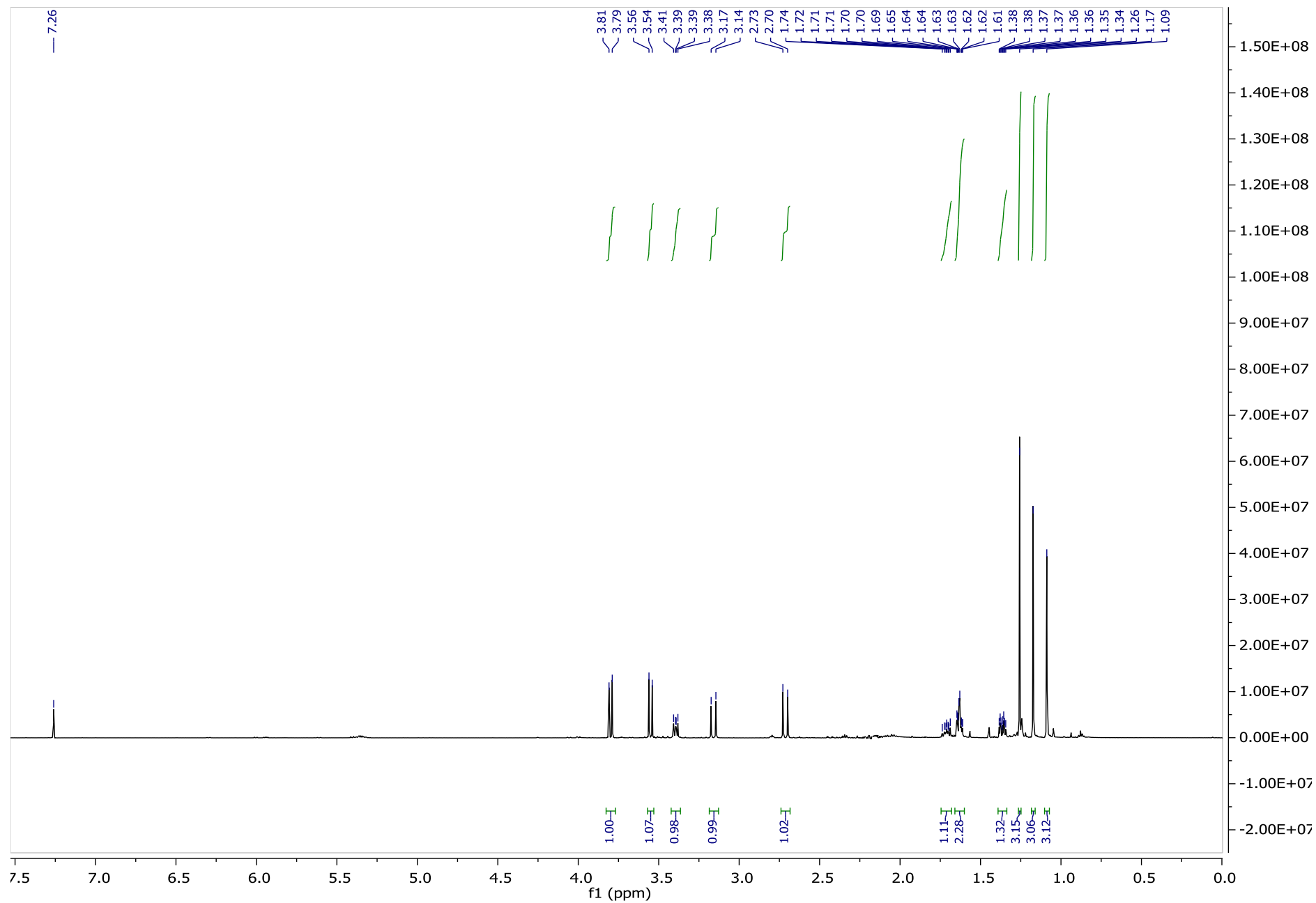

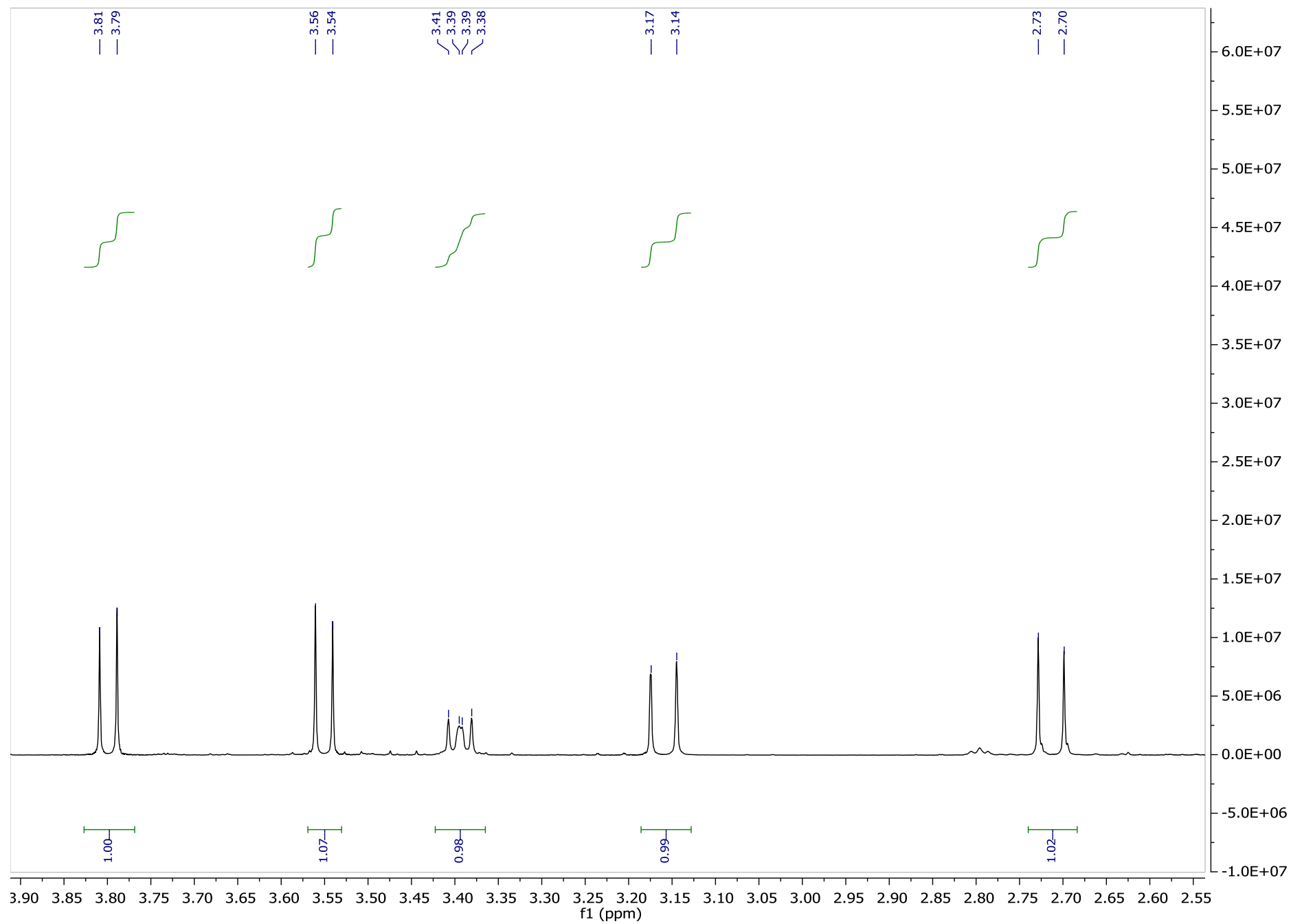

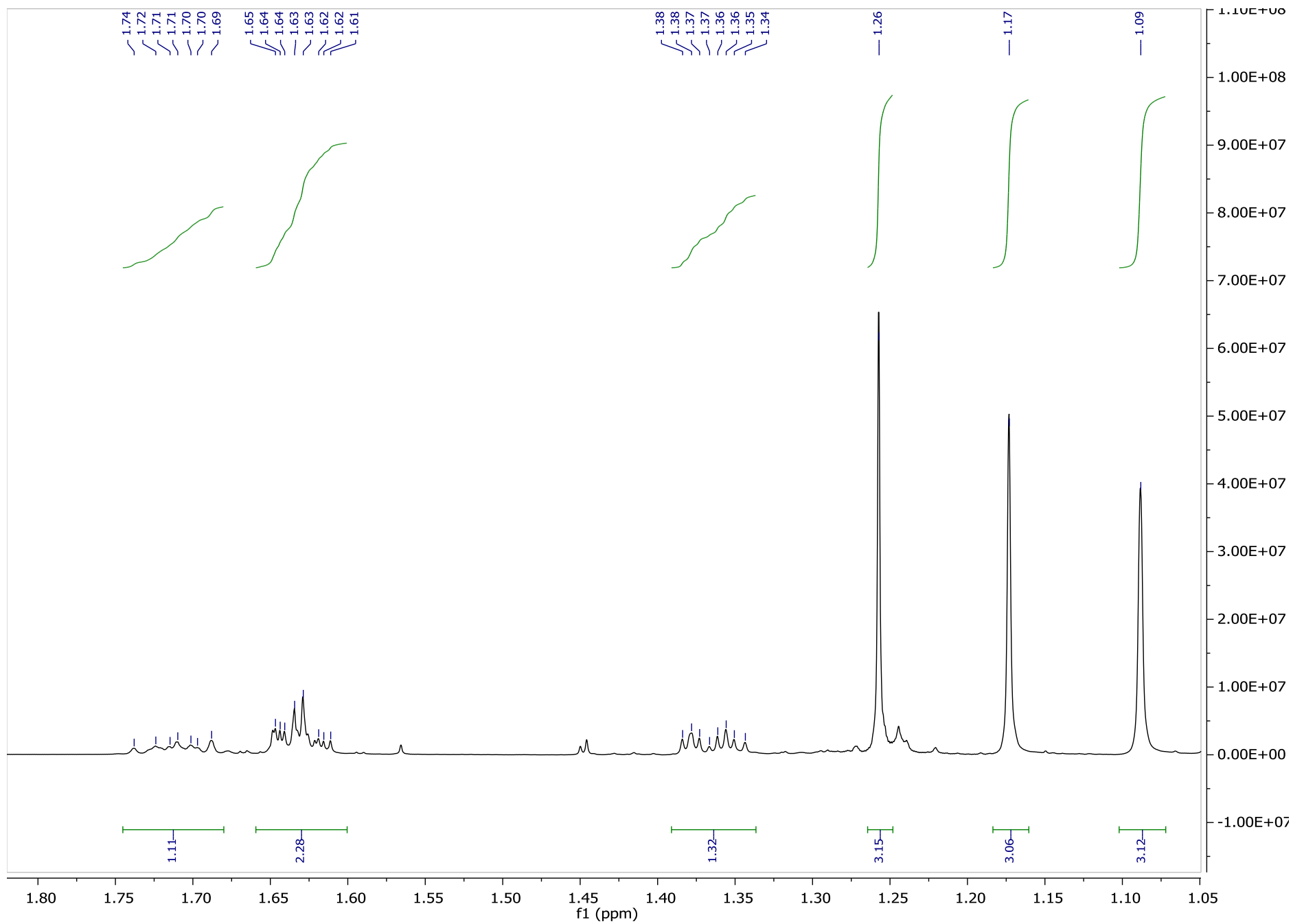

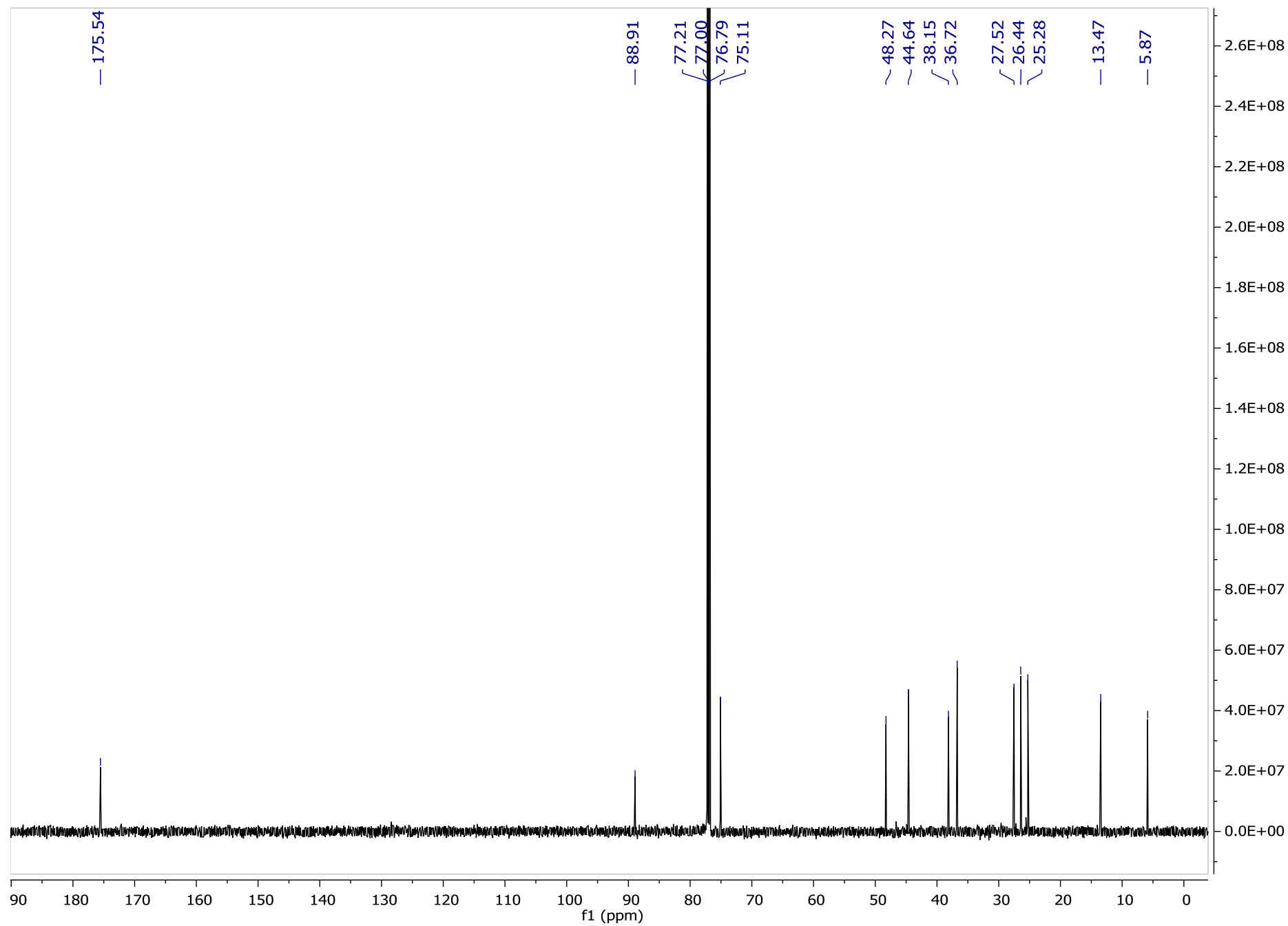

Supplement: S1 Fig — (PDF) [file pone.0183429.s001.pdf]

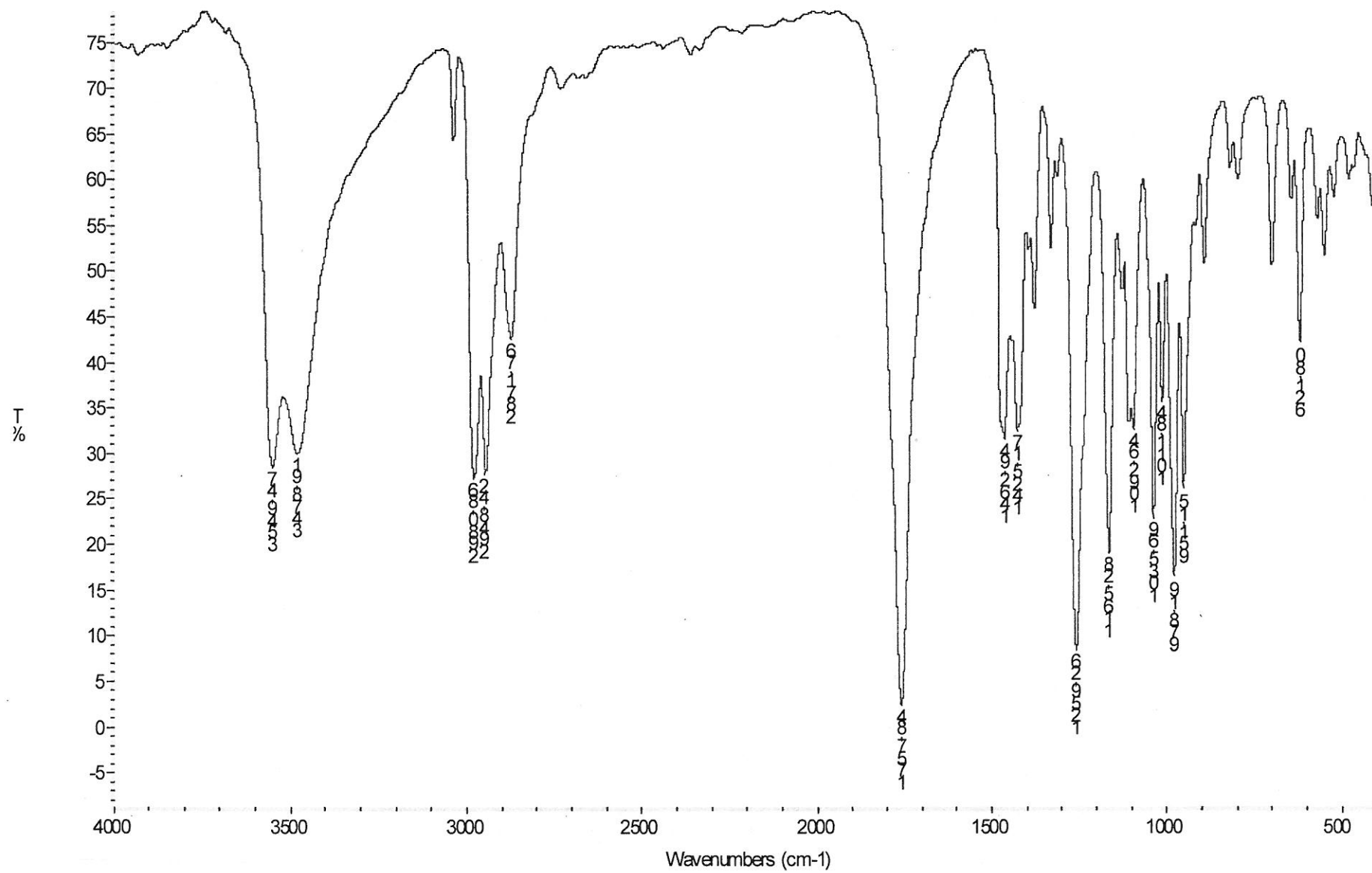

Product 4

Supplement: S2 Fig — (PDF) [file pone.0183429.s002.pdf]

Product 5

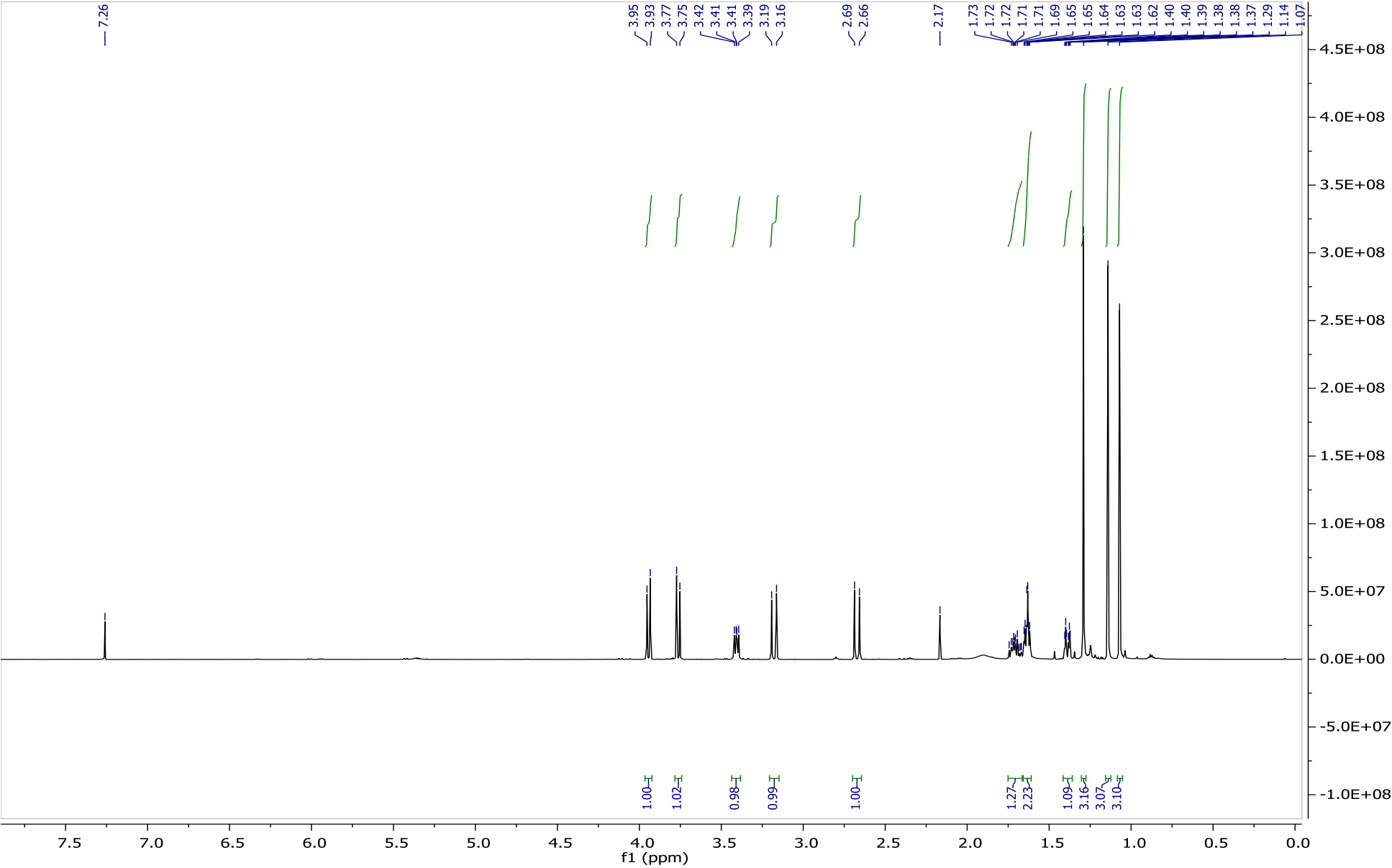

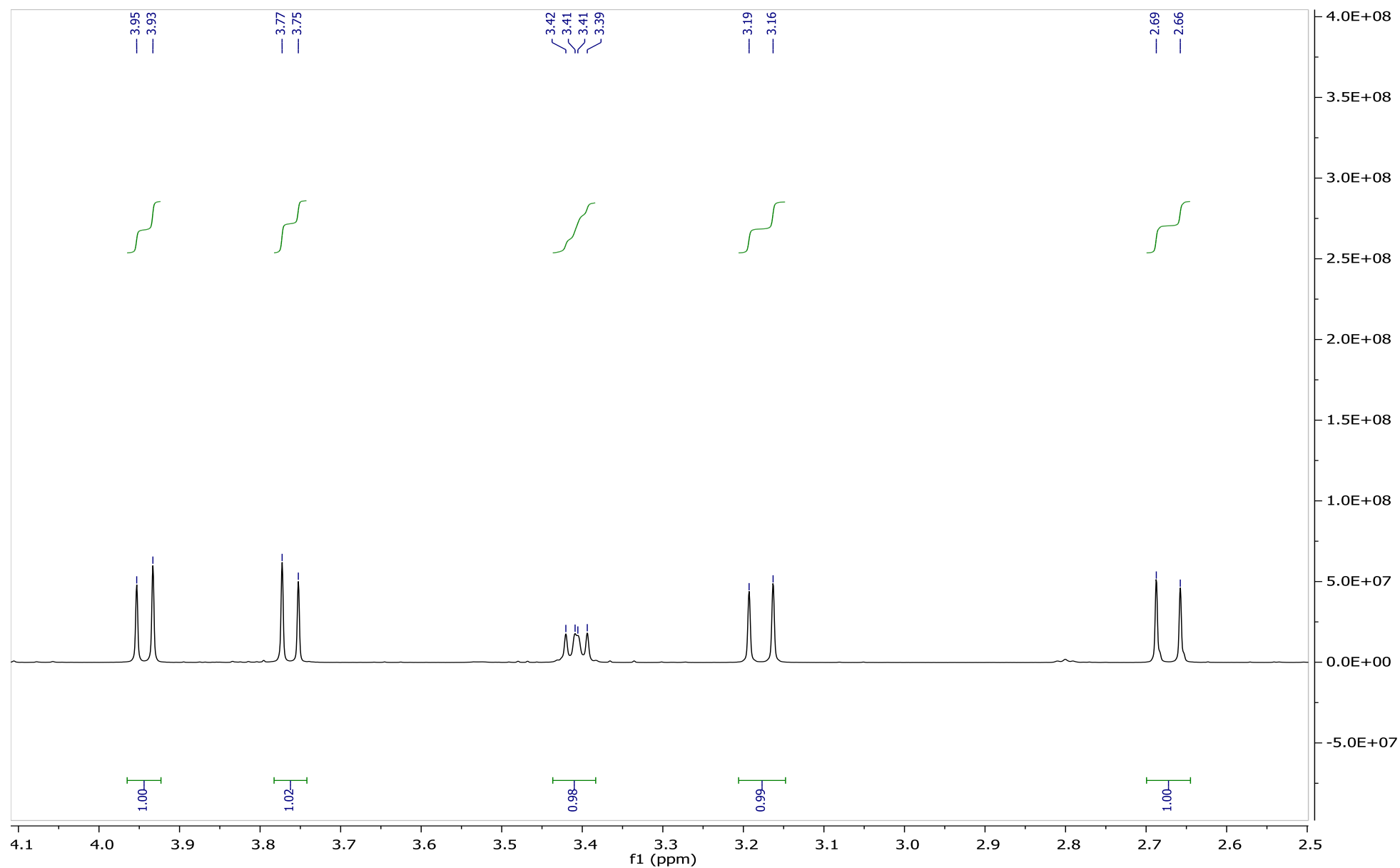

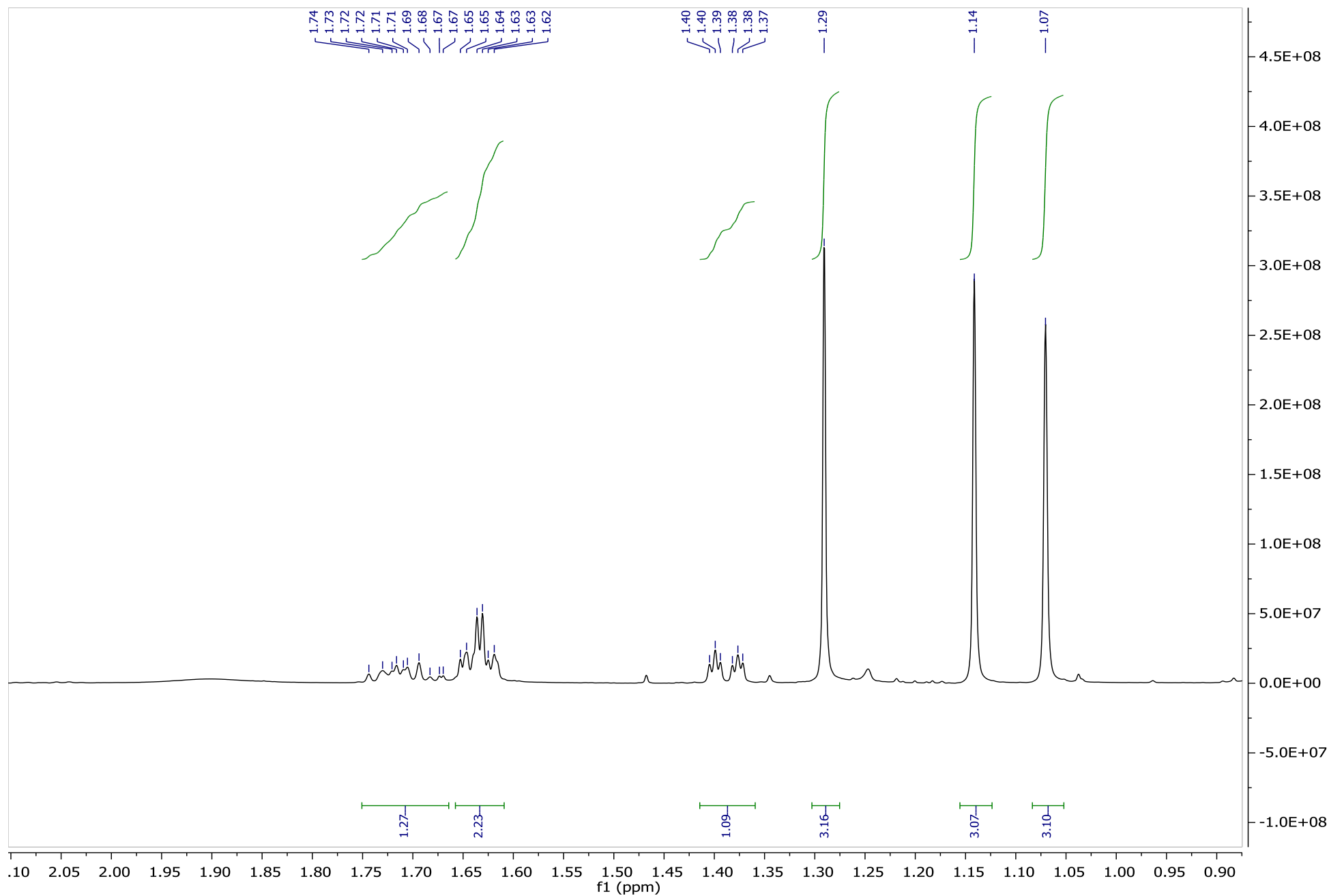

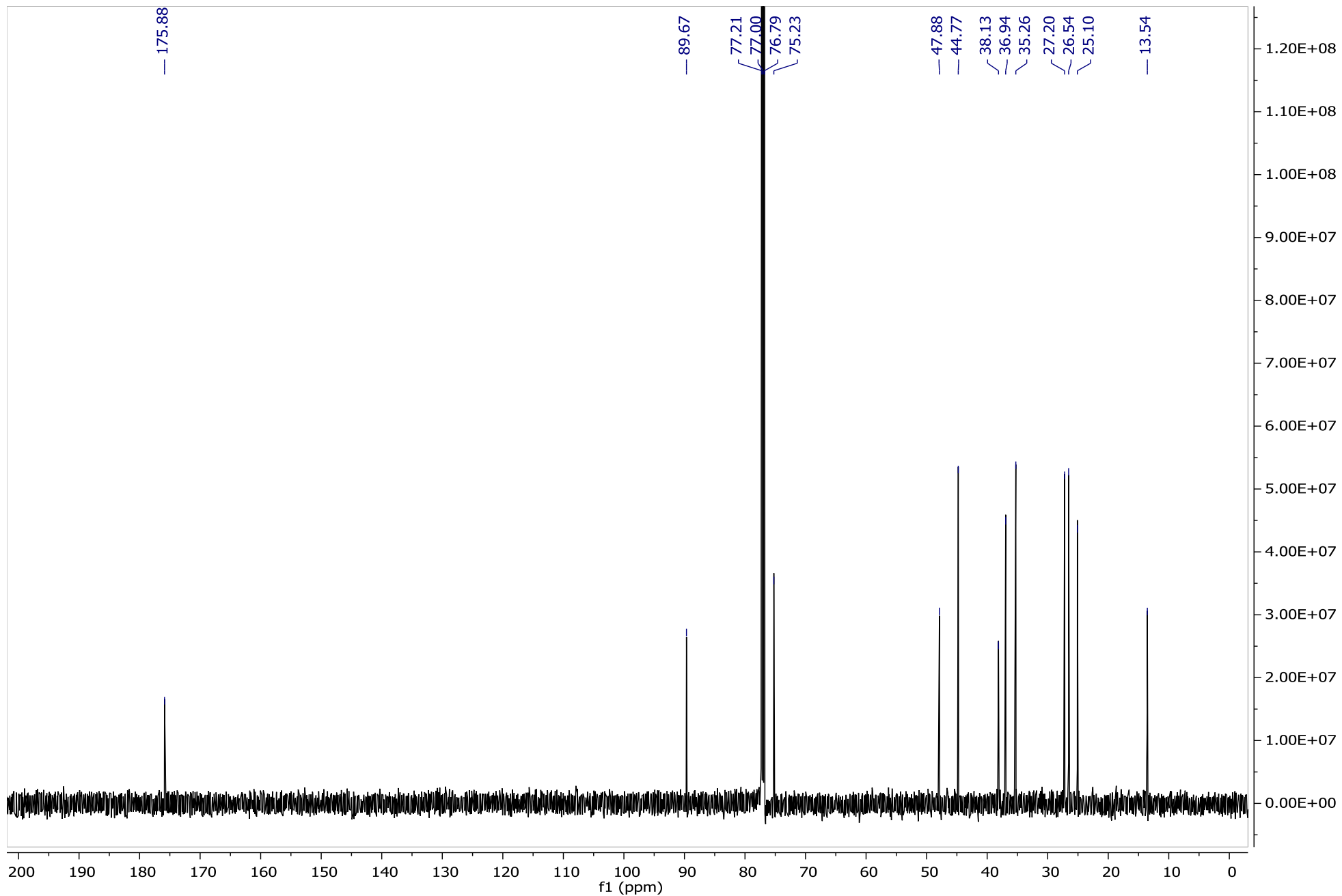

Supplement: S4 Fig — (PDF) [file pone.0183429.s004.pdf]

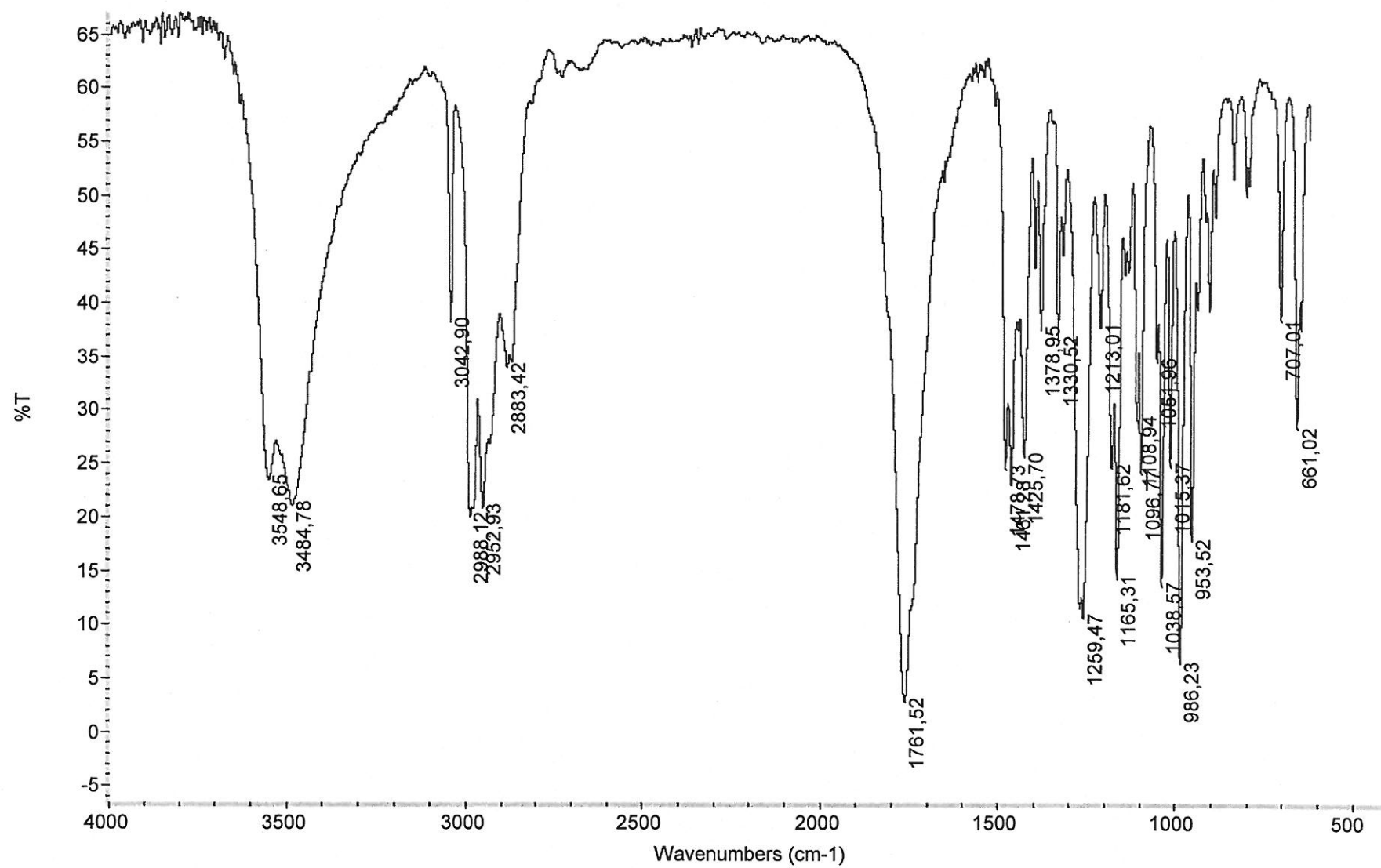

Product 5

Supplement: S5 Fig — (PDF) [file pone.0183429.s005.pdf]

Product 7

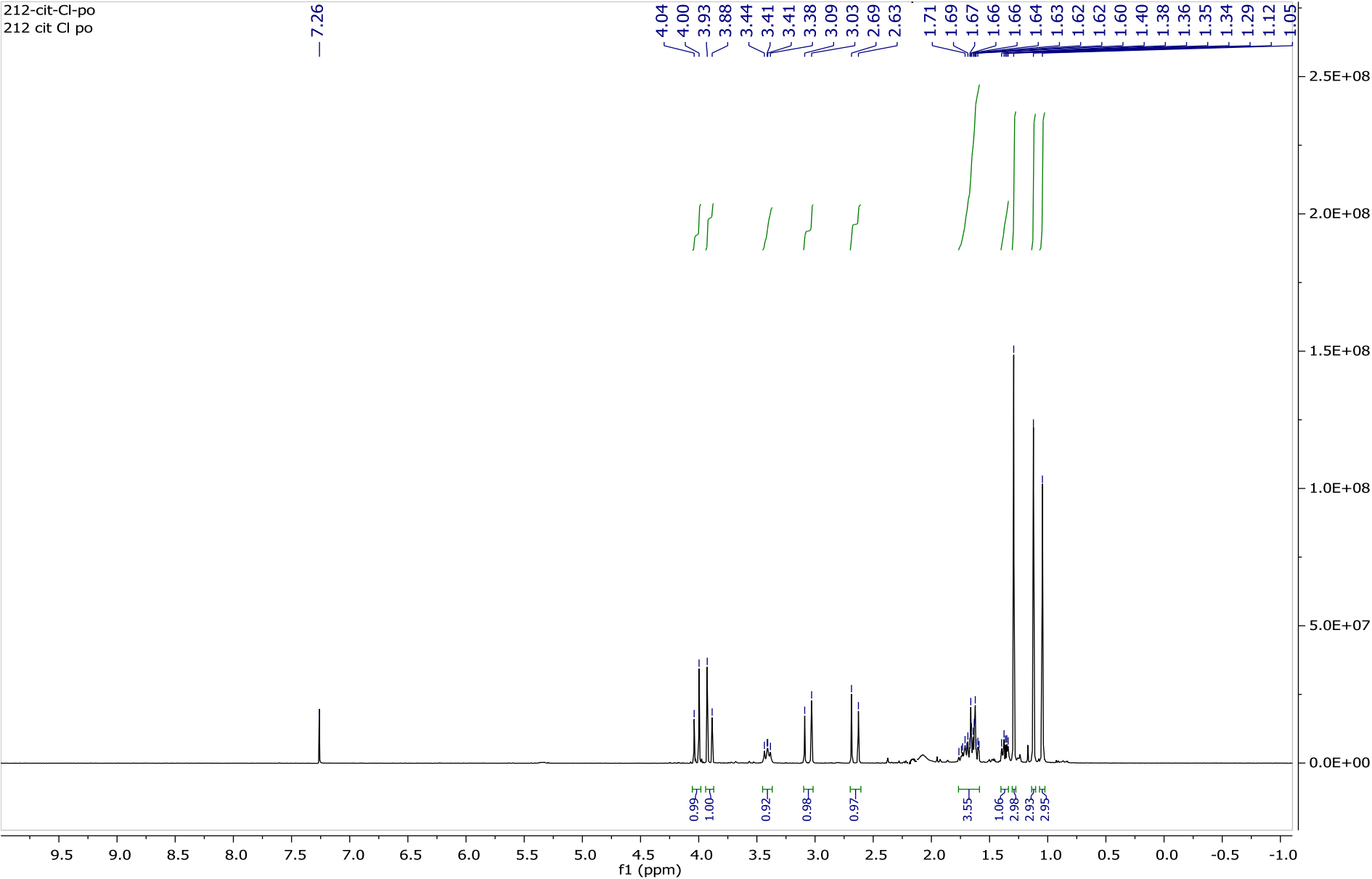

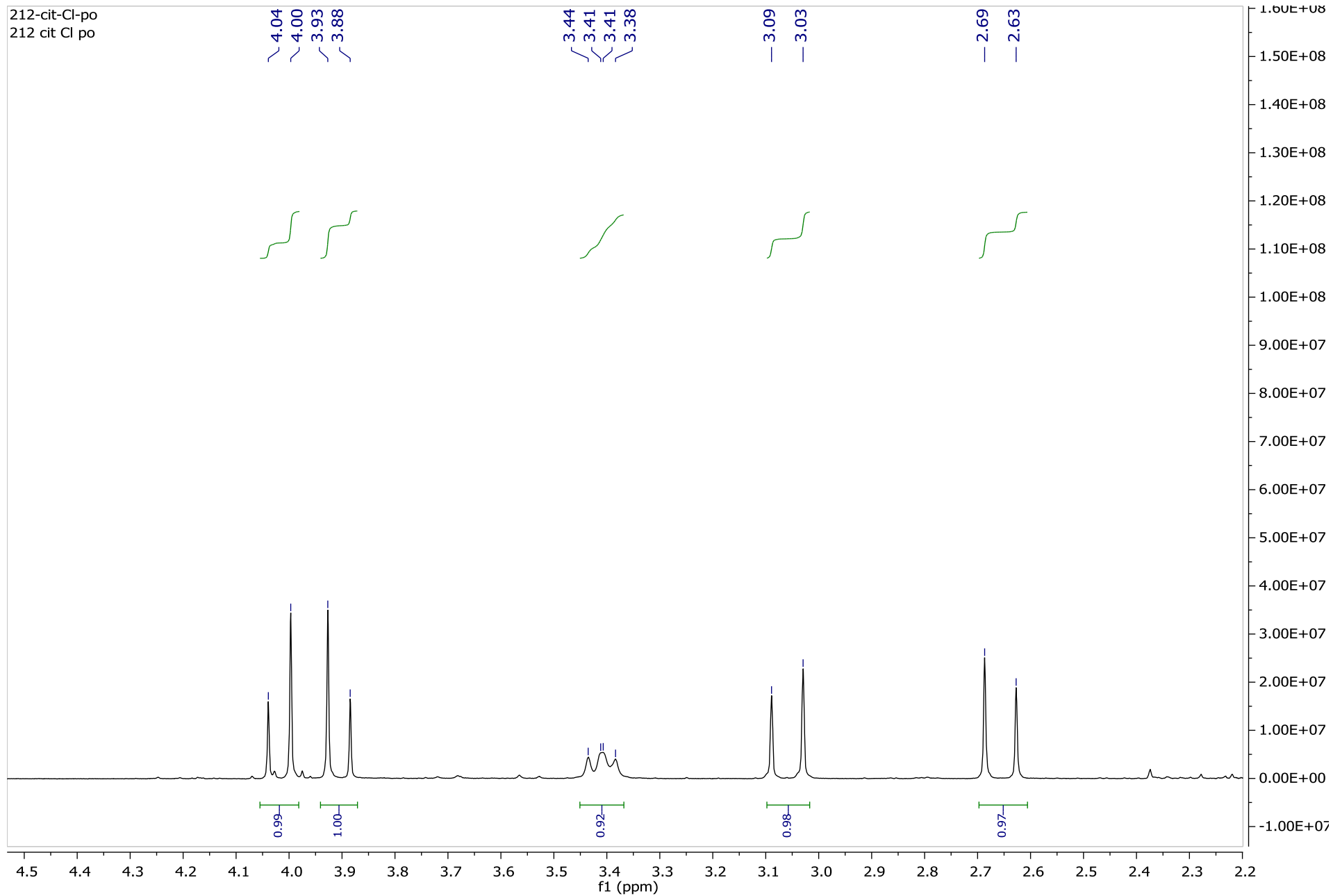

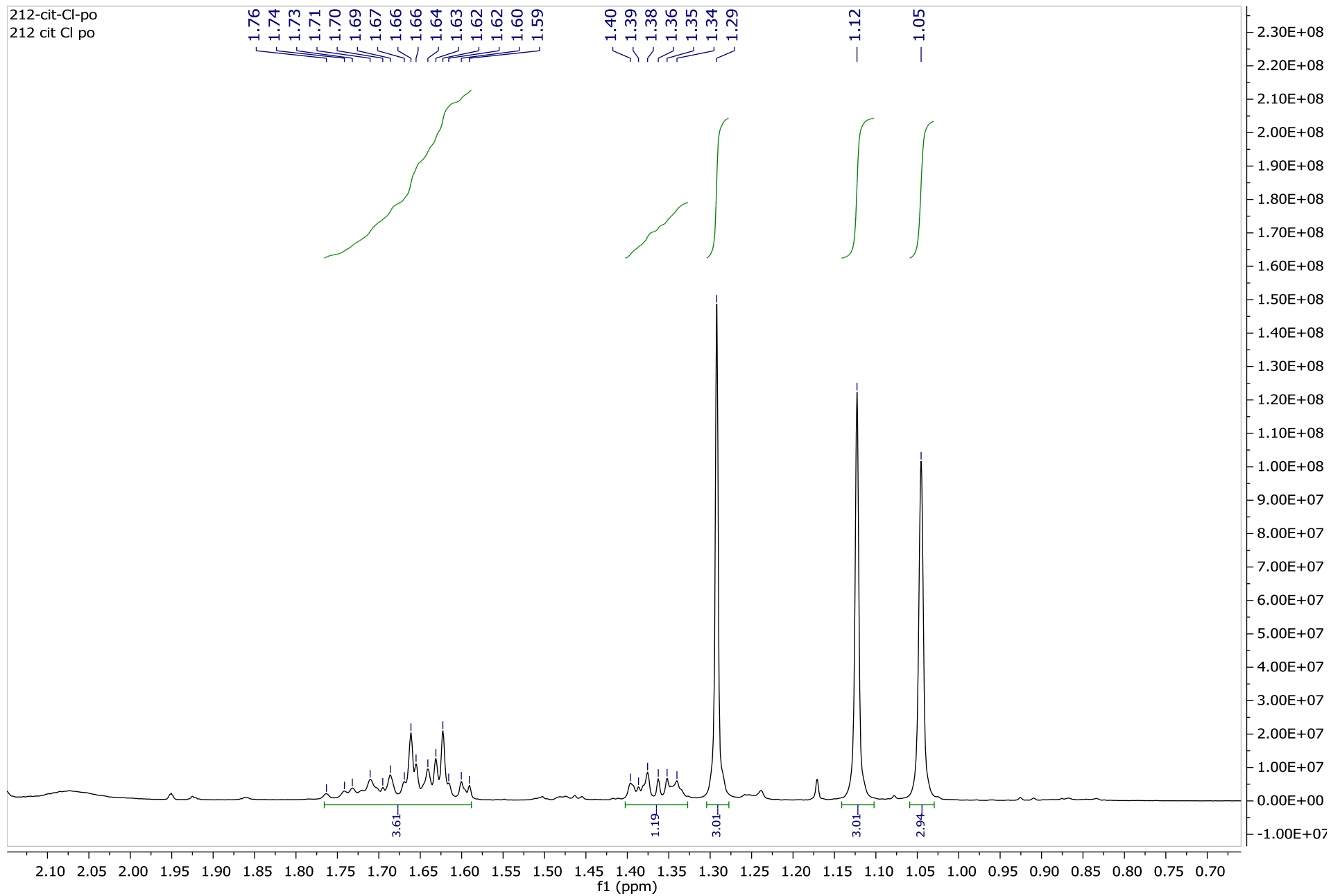

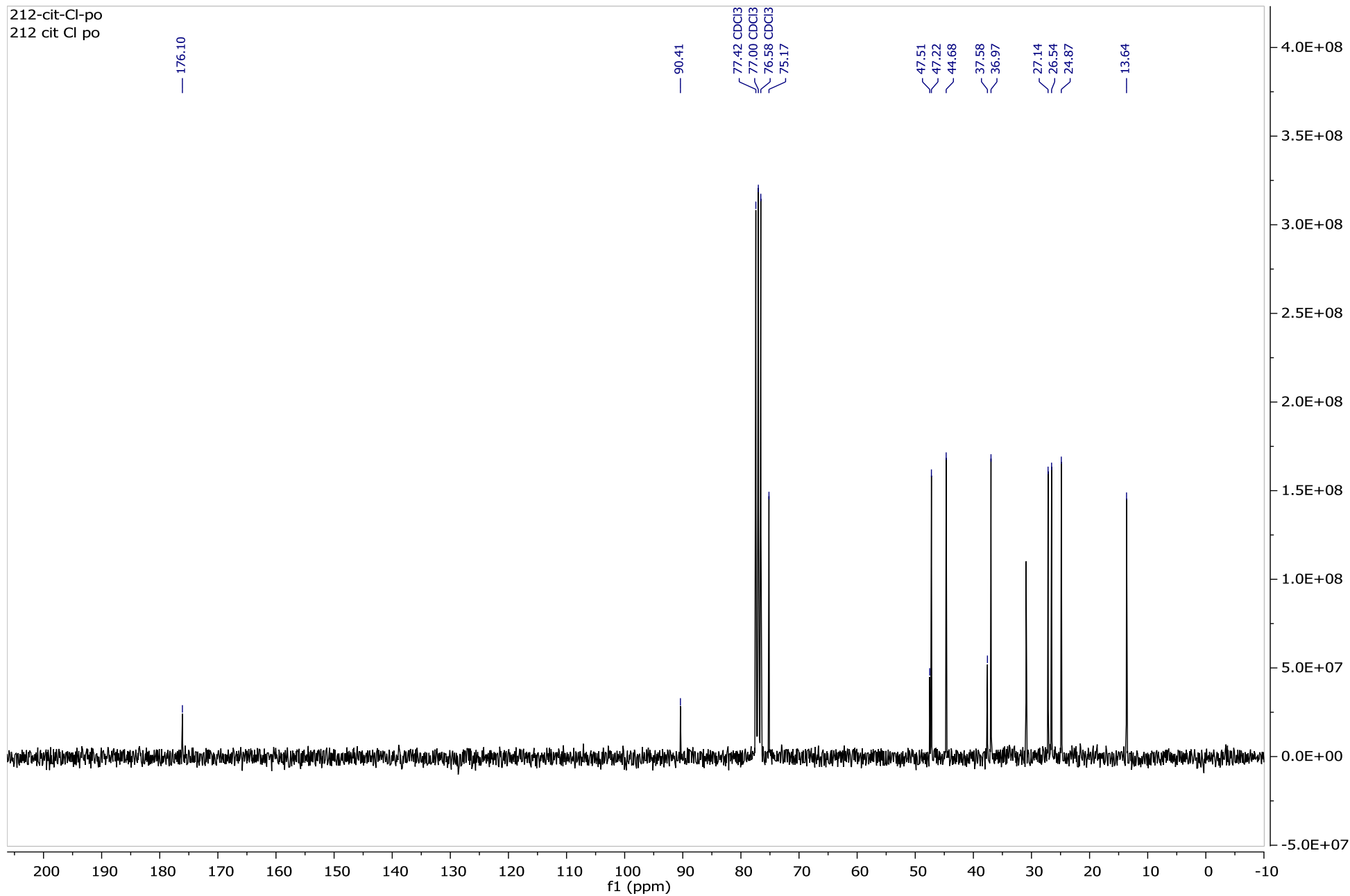

Supplement: S7 Fig — (PDF) [file pone.0183429.s007.pdf]

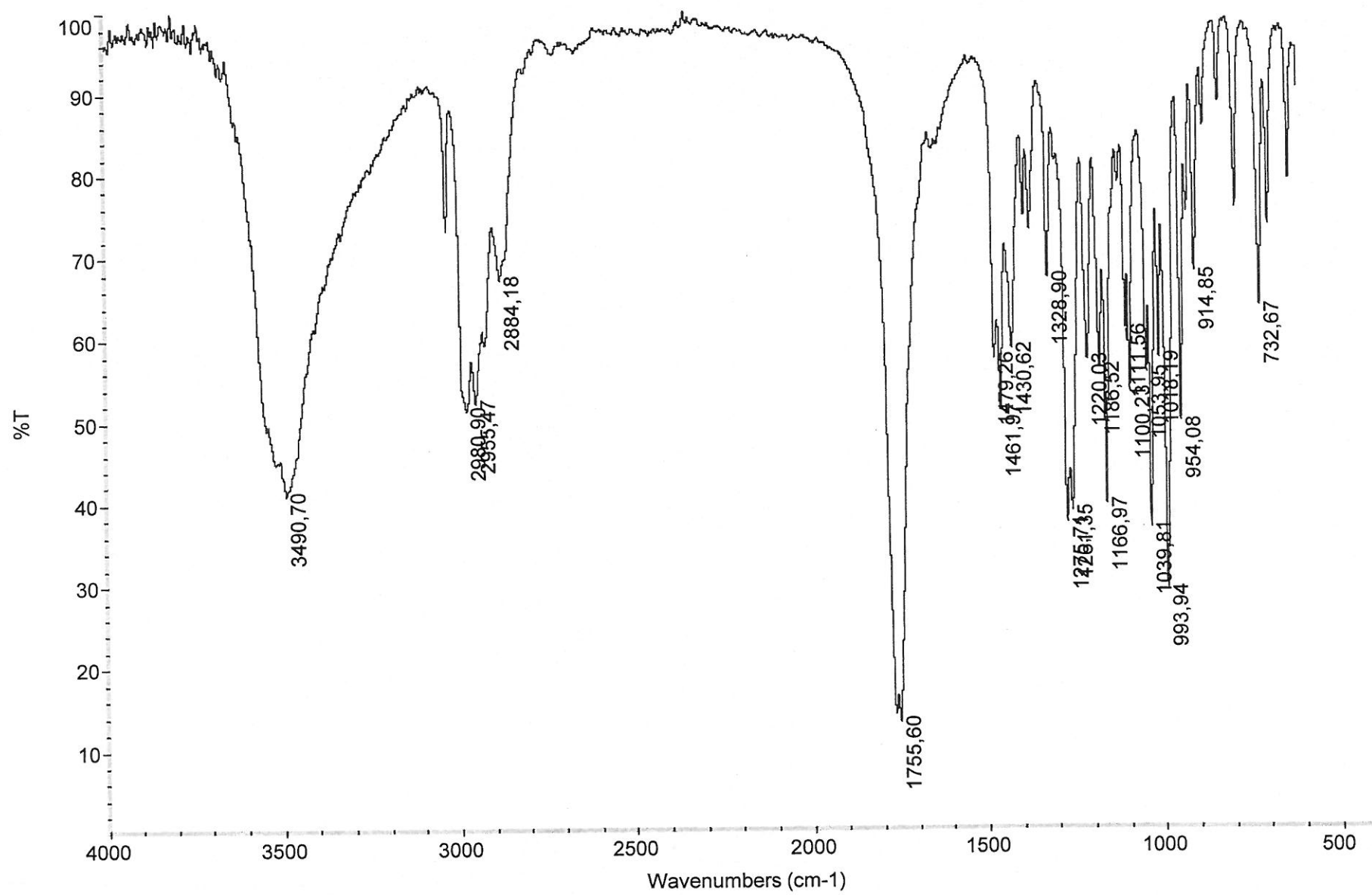

Product 7

Supplement: S8 Fig — (PDF) [file pone.0183429.s008.pdf]

Product 6

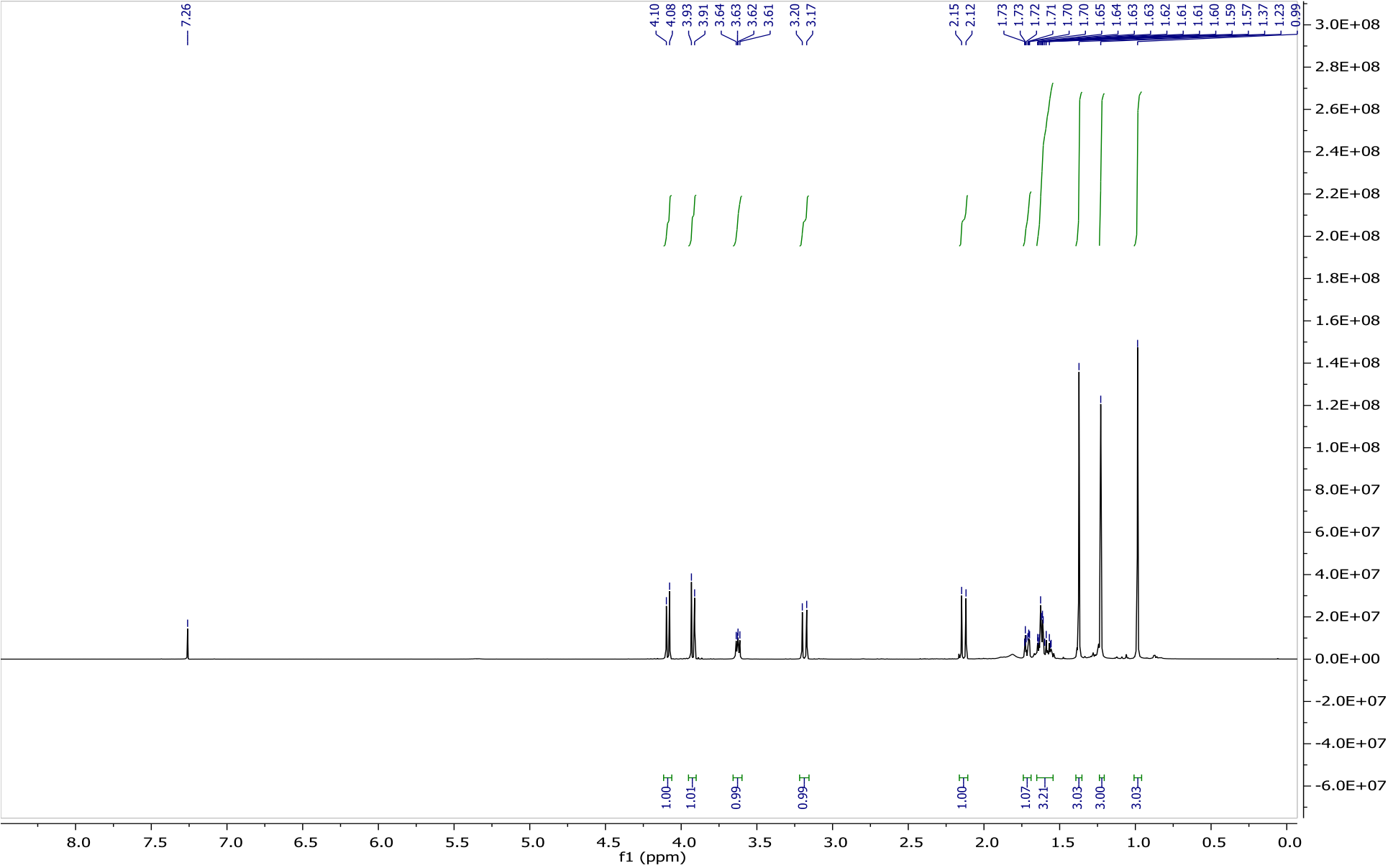

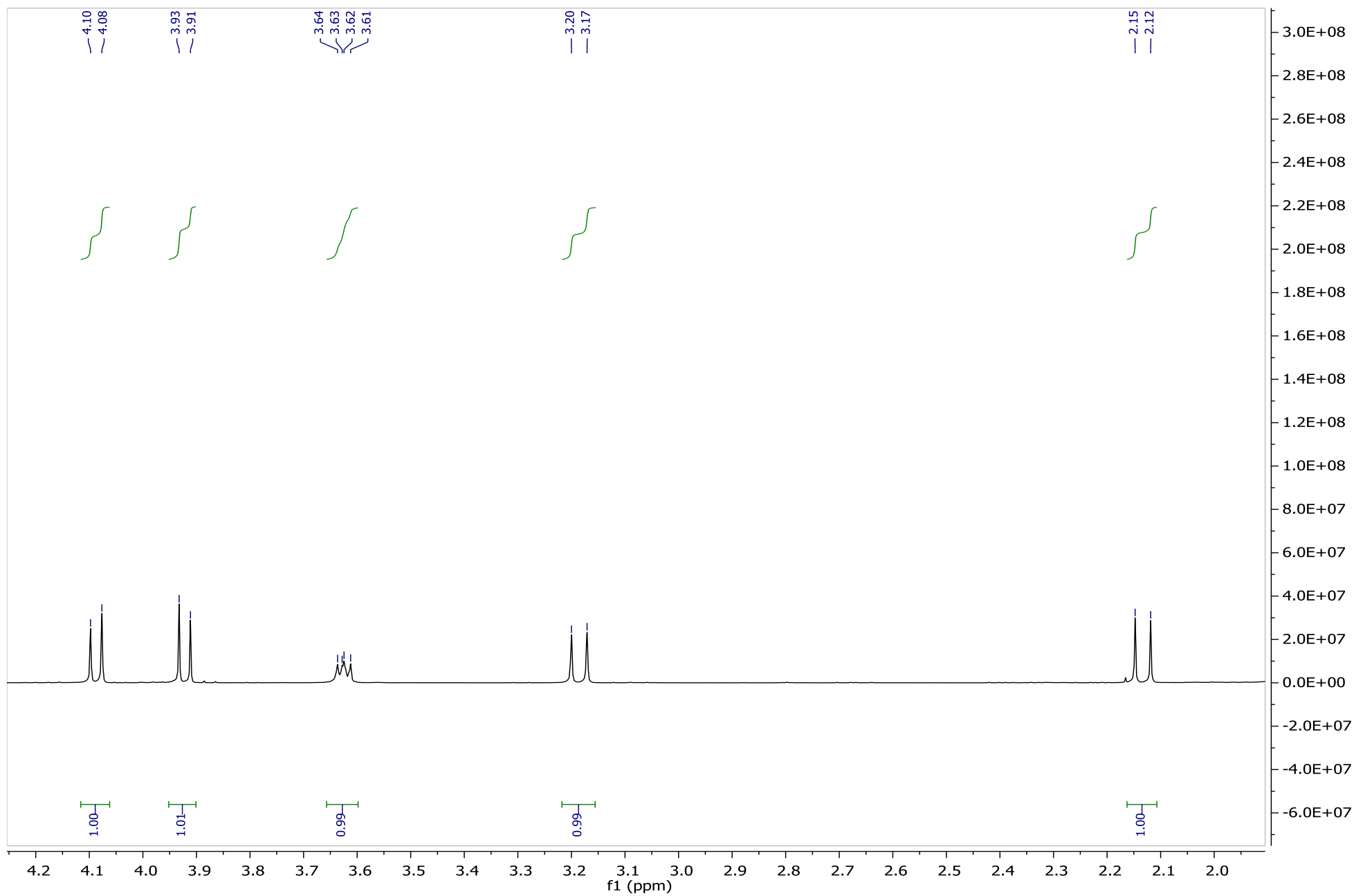

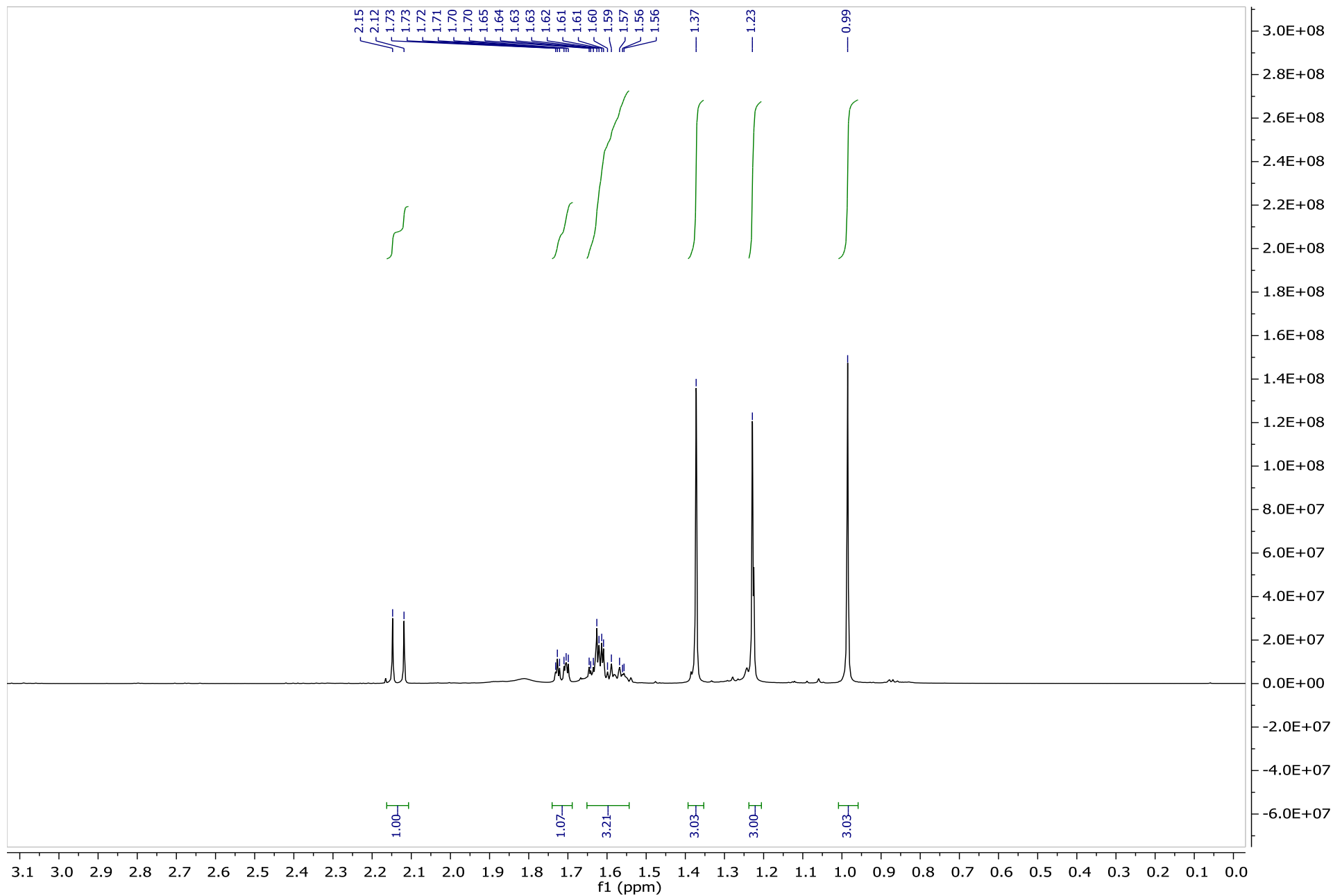

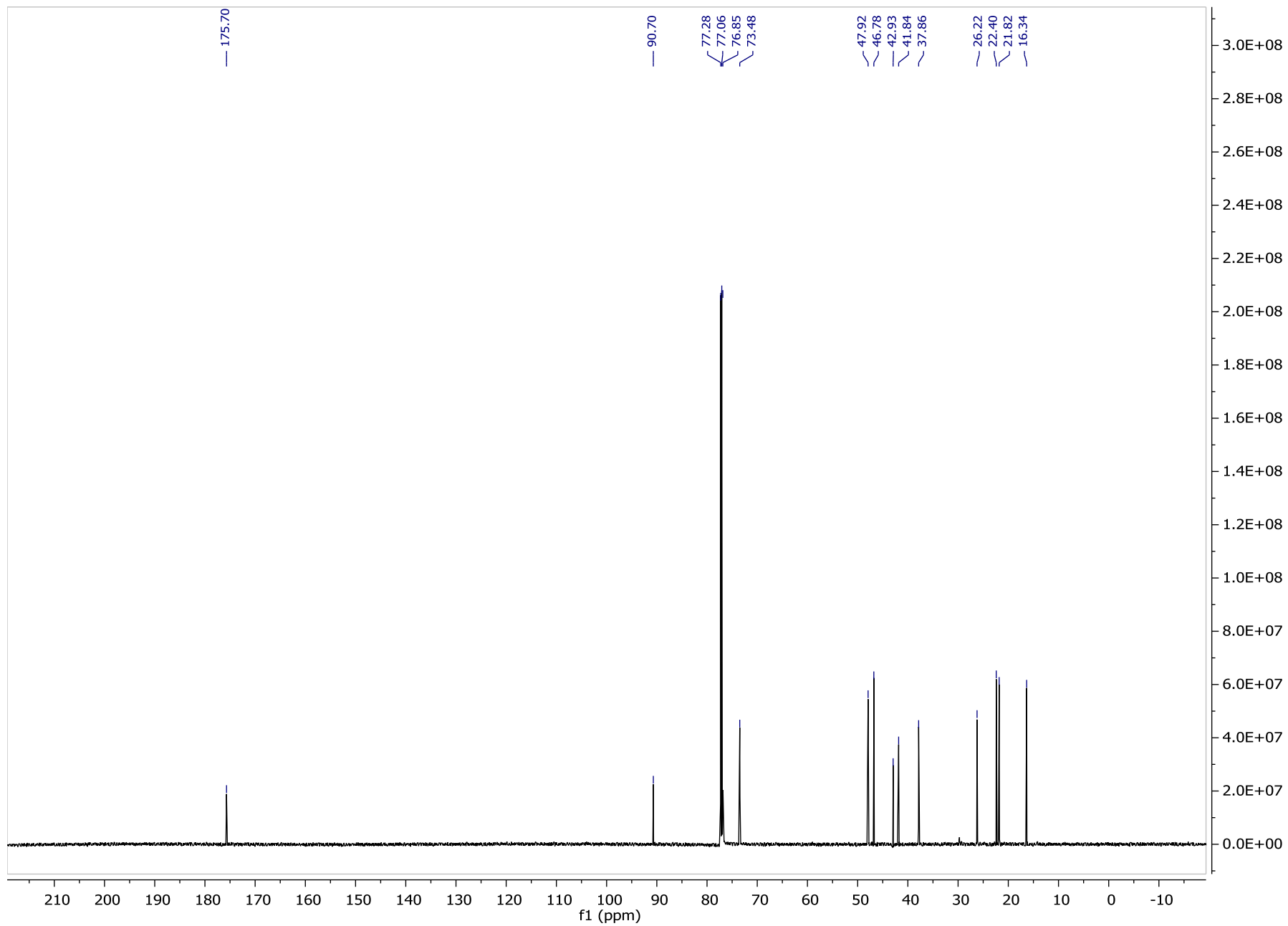

Supplement: S10 Fig — (PDF) [file pone.0183429.s010.pdf]

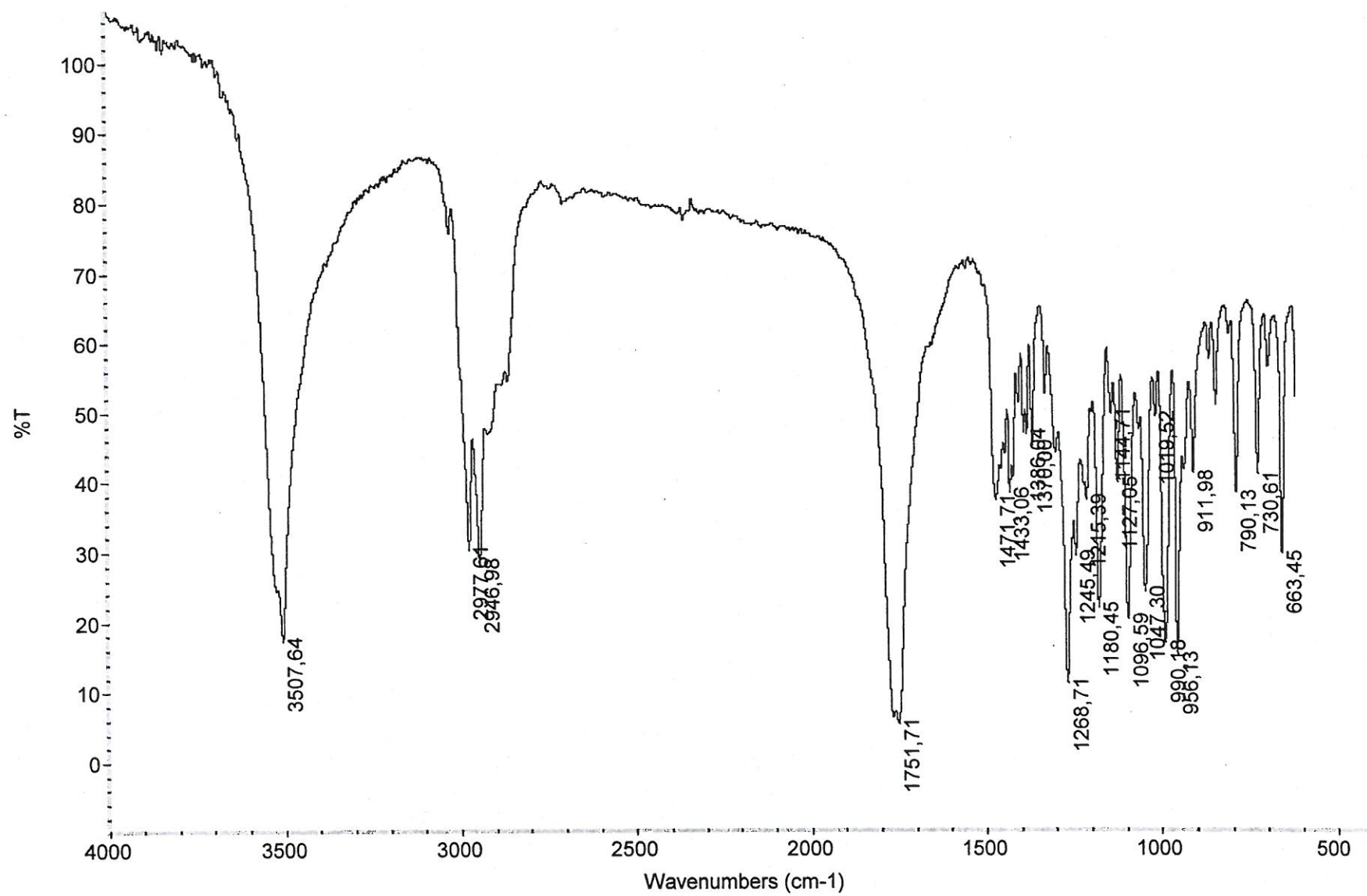

Product 6

Supplement: S11 Fig — (PDF) [file pone.0183429.s011.pdf]
