# Supplementary material for: Regio- and enantioselective microbial hydroxylation and evaluation of cytotoxic activity of β-cyclocitral-derived halolactones
Source: PLoS One. 2017 Aug 24;12(8):e0183429. doi: 10.1371/journal.pone.0183429 (PMC5570294; doi:10.1371/journal.pone.0183429)
Supplement: S3 Fig — (PDF) [file pone.0183429.s003.pdf]

Product 4

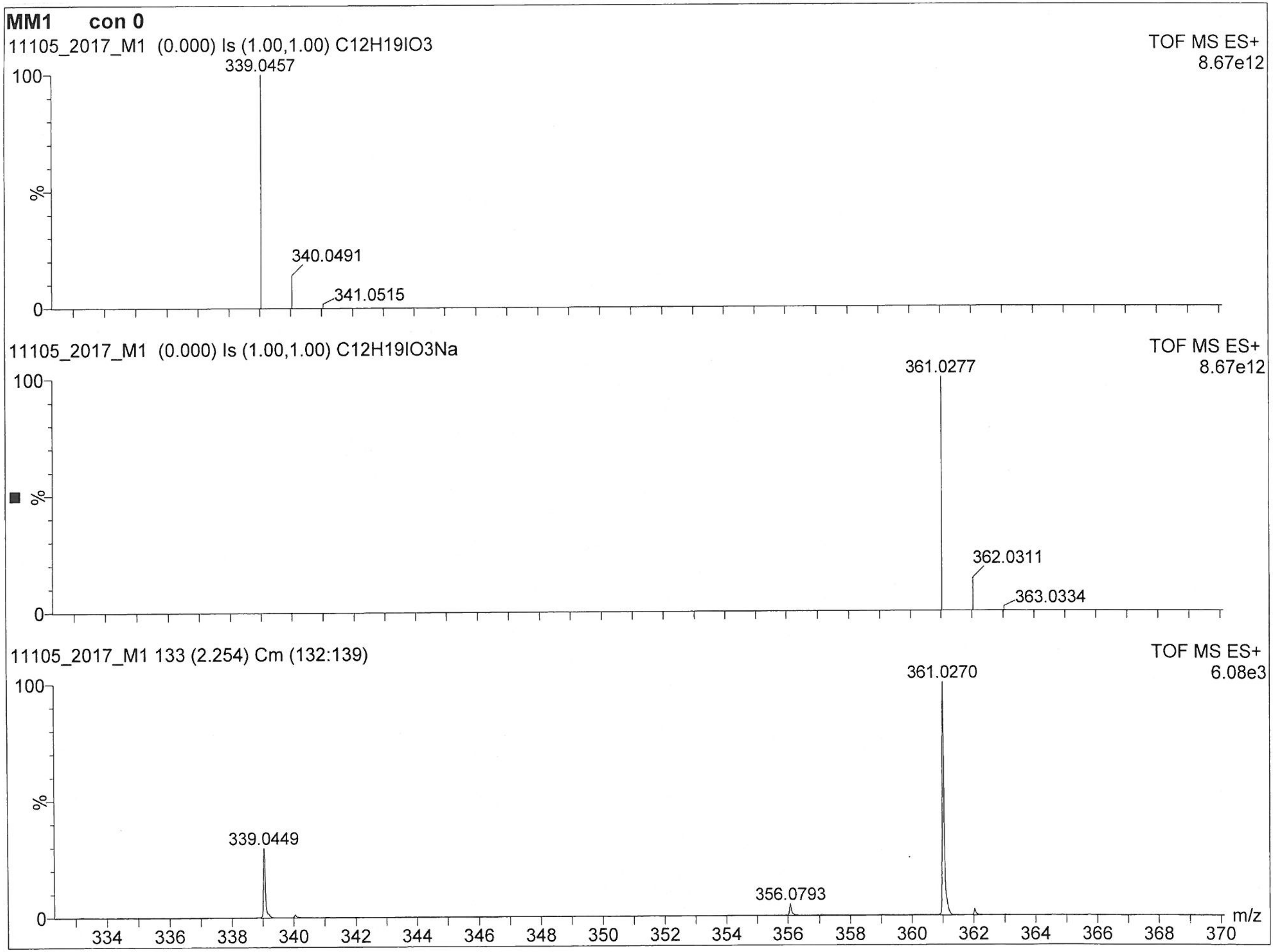
MM1 con 0
11105_2017_M1 (0.000) Is (1.00,1.00) C12H19IO3
TOF MS ES+
8.67e12
339.0457
340.0491
341.0515
11105_2017_M1 (0.000) Is (1.00,1.00) C12H19IO3Na
TOF MS ES+
8.67e12
361.0277
362.0311
363.0334
11105_2017_M1 133 (2.254) Cm (132:139)
TOF MS ES+
6.08e3
361.0270
339.0449
356.0793
%
100
0
m/z
334 336 338 340 342 344 346 348 350 352 354 356 358 360 362 364 366 368 370
