# Supplementary material for: Regio- and enantioselective microbial hydroxylation and evaluation of cytotoxic activity of β-cyclocitral-derived halolactones
Source: PLoS One. 2017 Aug 24;12(8):e0183429. doi: 10.1371/journal.pone.0183429 (PMC5570294; doi:10.1371/journal.pone.0183429)
Supplement: S6 Fig — (PDF) [file pone.0183429.s006.pdf]

Product 5

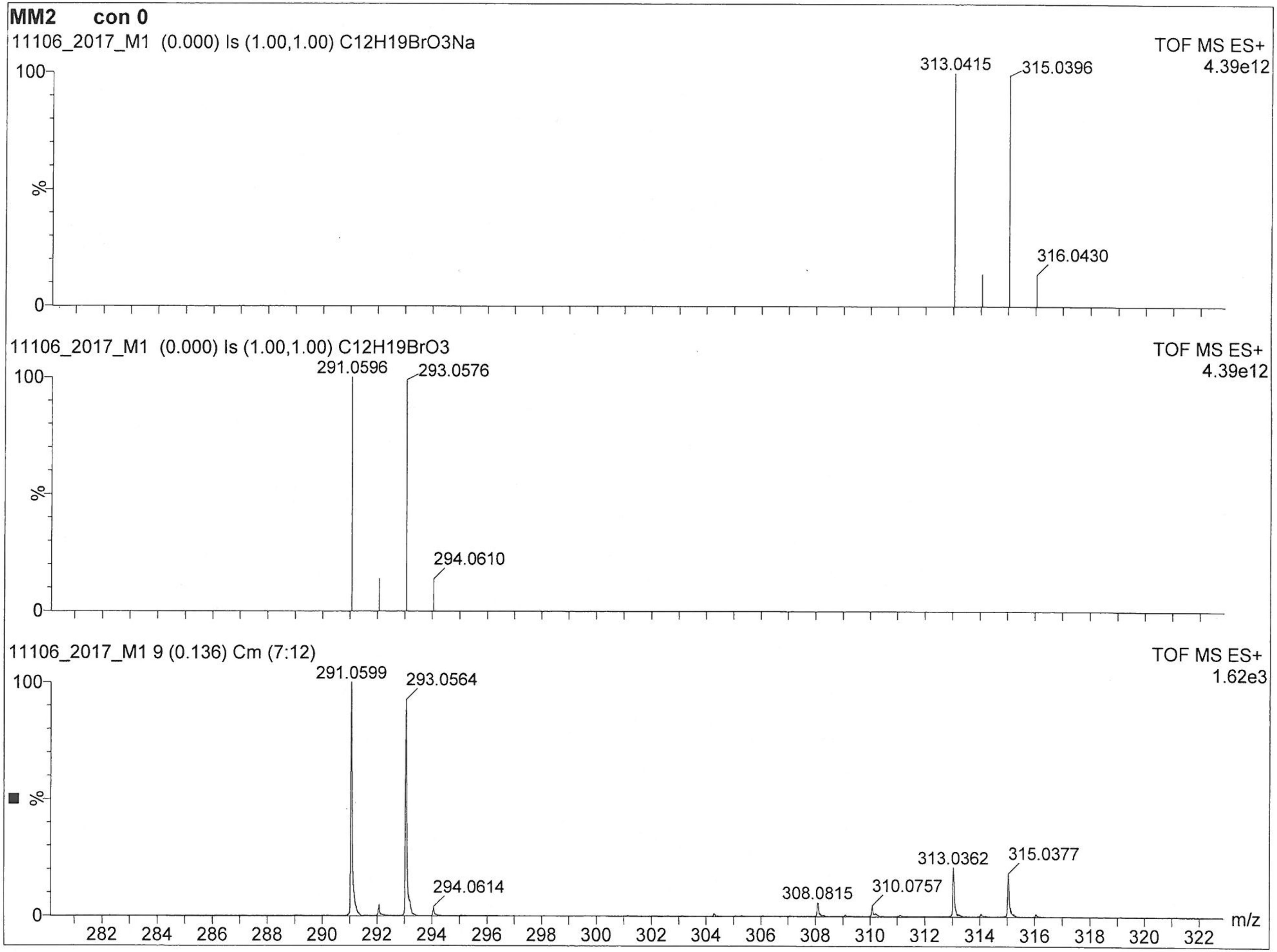

MM2 con 0
11106_2017_M1 (0.000) Is (1.00,1.00) C12H19BrO3Na
TOF MS ES+
4.39e12
313.0415
315.0396
316.0430
100
%
0
11106_2017_M1 (0.000) Is (1.00,1.00) C12H19BrO3
TOF MS ES+
4.39e12
291.0596
293.0576
294.0610
11106_2017_M1 9 (0.136) Cm (7:12)
TOF MS ES+
1.62e3
291.0599
293.0564
294.0614
308.0815
310.0757
313.0362
315.0377
282 284 286 288 290 292 294 296 298 300 302 304 306 308 310 312 314 316 318 320 322
m/z
