# Supplementary material for: Regio- and enantioselective microbial hydroxylation and evaluation of cytotoxic activity of β-cyclocitral-derived halolactones
Source: PLoS One. 2017 Aug 24;12(8):e0183429. doi: 10.1371/journal.pone.0183429 (PMC5570294; doi:10.1371/journal.pone.0183429)
Supplement: S9 Fig — (PDF) [file pone.0183429.s009.pdf]

Product 7

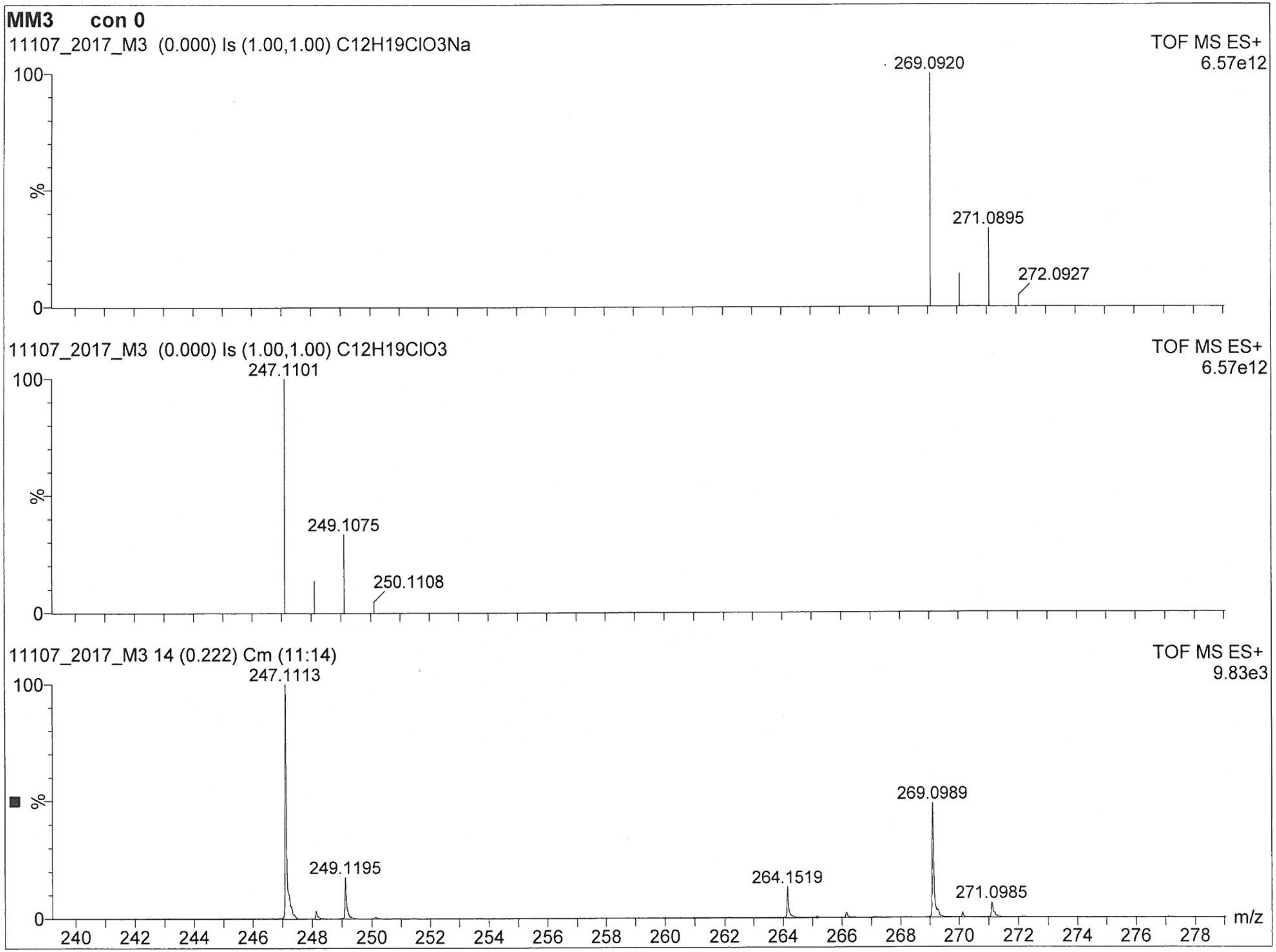
MM3 con 0
11107_2017_M3 (0.000) Is (1.00,1.00) C12H19ClO3Na
TOF MS ES+
6.57e12
269.0920
271.0895
272.0927
11107_2017_M3 (0.000) Is (1.00,1.00) C12H19ClO3
TOF MS ES+
6.57e12
247.1101
249.1075
250.1108
11107_2017_M3 14 (0.222) Cm (11:14)
TOF MS ES+
9.83e3
247.1113
249.1195
264.1519
269.0989
271.0985
100
%
0
m/z
240 242 244 246 248 250 252 254 256 258 260 262 264 266 268 270 272 274 276 278
