# Supplementary material for: Regio- and enantioselective microbial hydroxylation and evaluation of cytotoxic activity of β-cyclocitral-derived halolactones
Source: PLoS One. 2017 Aug 24;12(8):e0183429. doi: 10.1371/journal.pone.0183429 (PMC5570294; doi:10.1371/journal.pone.0183429)
Supplement: S12 Fig — (PDF) [file pone.0183429.s012.pdf]

Product 6

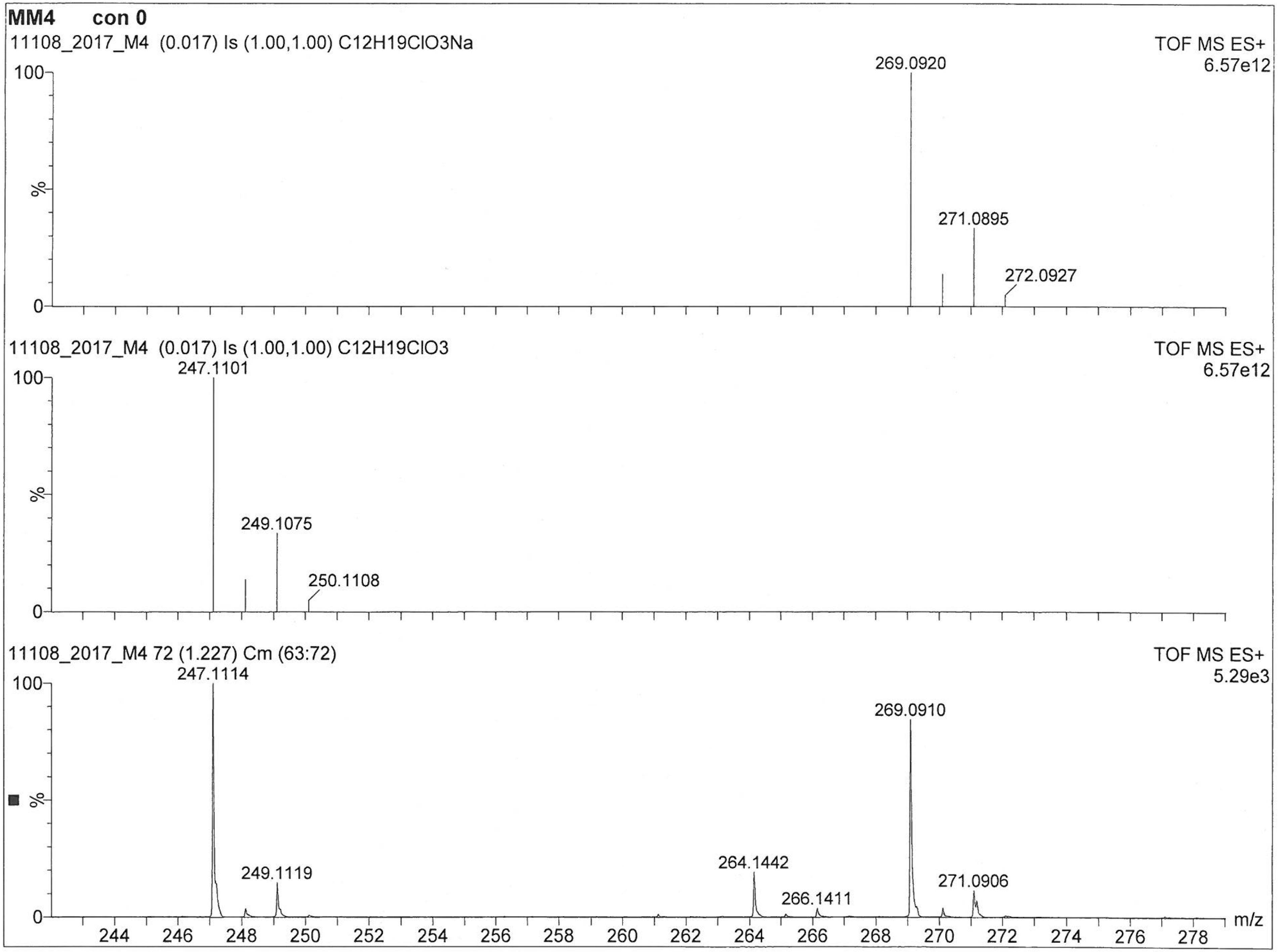
MM4 con 0
11108_2017_M4 (0.017) Is (1.00,1.00) C12H19ClO3Na
TOF MS ES+
6.57e12
269.0920
271.0895
272.0927
11108_2017_M4 (0.017) Is (1.00,1.00) C12H19ClO3
TOF MS ES+
6.57e12
247.1101
249.1075
250.1108
11108_2017_M4 72 (1.227) Cm (63:72)
TOF MS ES+
5.29e3
247.1114
269.0910
249.1119
264.1442
266.1411
271.0906
100
%
0
m/z
244 246 248 250 252 254 256 258 260 262 264 266 268 270 272 274 276 278
